# Imaging Biomarkers in Animal Models of Drug-Induced Lung Injury: A Systematic Review

**DOI:** 10.3390/jcm10010107

**Published:** 2020-12-30

**Authors:** Irma Mahmutovic Persson, Karin von Wachenfeldt, John C. Waterton, Lars E. Olsson, on behalf of the TRISTAN Consortium

**Affiliations:** 1 Department of Translational Medicine, Medical Radiation Physics, Lund University, 20502 Malmö, Sweden; lars_e.olsson@med.lu.se; 2 Truly Labs, Medicon Village, 223 63 Lund, Sweden; karin@trulylabs.com; 3 Bioxydyn Ltd., Science Park, Manchester M15 6SZ, UK; John.Waterton@bioxydyn.com; 4 Manchester Academic Health Sciences Centre, University of Manchester, Manchester M13 9PL, UK

**Keywords:** lung injury models, imaging biomarkers, magnetic resonance imaging (MRI), drug toxicity, lung imaging, computed tomography (CT), positron emission tomography (PET), drug-induced interstitial lung disease (DIILD), fibrosis, inflammation, vascular leak

## Abstract

For drug-induced interstitial lung disease (DIILD) translational imaging biomarkers are needed to improve detection and management of lung injury and drug-toxicity. Literature was reviewed on animal models in which in vivo imaging was used to detect and assess lung lesions that resembled pathological changes found in DIILD, such as inflammation and fibrosis. A systematic search was carried out using three databases with key words “Animal models”, “Imaging”, “Lung disease”, and “Drugs”. A total of 5749 articles were found, and, based on inclusion criteria, 284 papers were selected for final data extraction, resulting in 182 out of the 284 papers, based on eligibility. Twelve different animal species occurred and nine various imaging modalities were used, with two-thirds of the studies being longitudinal. The inducing agents and exposure (dose and duration) differed from non-physiological to clinically relevant doses. The majority of studies reported other biomarkers and/or histological confirmation of the imaging results. Summary of radiotracers and examples of imaging biomarkers were summarized, and the types of animal models and the most used imaging modalities and applications are discussed in this review. Pathologies resembling DIILD, such as inflammation and fibrosis, were described in many papers, but only a few explicitly addressed drug-induced toxicity experiments.

## 1. Introduction

Drug-induced interstitial lung disease (DIILD) covers a range of pathological states that may occur in patients after exposure to various investigational or approved drugs [1], mostly administered systemically and not by inhalation. Bleomycin (anti-neoplastic), nitrofurantoin (anti-infective), and amiodarone (anti-arrhythmic) are well-known examples [2,3]. Additional examples are tumor necrosis factor-alpha (TNF-α) inhibitors and methotrexate used for treating autoimmune or inflammatory diseases, such as rheumatoid arthritis, psoriasis, or Crohn’s disease [2,3], and, importantly, the recently developed checkpoint inhibitors in cancer treatment often induce DIILD [4]. DIILD is an increasing issue [2,4,5] as new drugs continuously enter the market [2,3,6].

For ILD, in general, there has been a classification by the International Multidisciplinary group from American Thoracic Society (ATS)/ European Respiratory Society (ERS), whereas DIILD was defined within the subgroup of known cause, being the drug-induced type of ILD [7]. Despite clear classification of DIILD, it is difficult to detect and distinguish it from other ILD conditions, given that drugs may give rise to very variable pathophysiology, while other underlying pulmonary disorders may interfere with the diagnosis of DIILD. When radiological assessments indicate an onset of ILD initiated upon drug administration, and other causes of disease are ruled out, DIILD should be suspected. Nonetheless, DIILD is not confirmed until reversibility upon drug removal is observed [7,8]. Most often, management of DIILD is to withdraw the suspected drug agent, and often additional prescription of glucocorticoids may help to reverse the inflammation [1,9]. However, some patients do not improve. They may instead continuously progress towards lung fibrosis and eventually lung failure [2,3,10]. Early biomarkers to identify patients who will not improve upon drug withdrawal might enable early treatment to prevent disease progression of DIILD. There are no generic pathological or radiological patterns for how this disease manifests itself. However, some drugs do manifest specific DIILD changes: for example, methotrexate is mostly associated with induction of non-specific interstitial pneumonia (NSIP) [2,11,12]. Presently, computed tomography (CT) is the radiological modality of choice for ILD in clinical applications. However, imaging modalities, such as magnetic resonance imaging (MRI) and positron emission tomography (PET), have shown substantial progress for diagnosis and further characterization of ILD [9,13,14,15,16,17]. Histopathologically, lung toxicity may manifest as pulmonary edema, alveolar hemorrhage, diffuse alveolar damage (DAD), bronchiolitis obliterans organizing pneumonia (BOOP), or usual interstitial pneumonia-like pattern (UIP). In some cases, there may be presence of diffuse cellular interstitial infiltrates, with or without granulomas [5]. Sometimes the pathological assessment indicates lymphocytic or eosinophilic interstitial pneumonia, so-called cellular NSIP. Histological findings that resemble UIP or NSIP without appreciable airway disease or parenchymal scarring have been suggested as one of the most commonly reported morphological patterns that are associated with pulmonary drug toxicities [18].

The increasing issue of drug-induced toxicity has recently been identified by the Innovative Medicines Initiative (IMI)-initiated consortium Translational Imaging in Drug Safety Assessment (TRISTAN). The aim of this consortium is to bring DIILD into the focus of drug development and develop imaging biomarkers (IBs) for DIILD diagnosis and progression [19]. From the drug safety perspective, drug developers would benefit from having robust and translational IBs to identify and monitor DIILD pre-marketing. Often uncommon side effect in Phase III trials normally can cause progress of an otherwise promising treatment to be abandoned late in drug development. This represents a futile investment, not only of the R&D resources to take an investigational drug through Phase III but also of the contribution of patients who volunteered for those clinical trials. Drug developers could use validated translational IBs to identify investigational drugs with DIILD liability, either so that they could be abandoned early in drug development, or alternatively to develop prescribing information to reduce the risk of DIILD.

An animal model for IB development should recapitulate relevant elements of ILD, and successive phases of disease progression and pathological patterns should be expressed. Typically, models exhibiting fibrosis model late ILD, while other models mimic the early inflammatory phase. Both pathologies are seen in bleomycin models, often used to model idiopathic pulmonary fibrosis (IPF). Bleomycin is commonly used in oncology and could serve as a lung injury inducing agent for preclinical DIILD. However, it does not model all manifestations of clinical DIILD nor is it optimal to express true pathological changes seen in IPF, although it is convenient because of its robust repeatability and reproducibility [20,21].

At present, there is no systematic compilation of IBs in the preclinical setting. In this systematic review, we performed a comprehensive literature search according to the Preferred Reporting Items for Systematic reviews and Meta-analyses (PRISMA) consensus guidelines [22]. The first search covered years of publications from 1970–2017. Additional updated search was performed to include latest articles published between 2017 and mid-2019 (July). Human studies were not included in this review. The aim of this systematic review was primarily to identify animal models of interstitial lung disease where non-invasive and in vivo imaging has been implemented. Therefore, we investigated the literature on in vivo imaging to detect and assess lung lesions in animal models with the potential to further develop and eventually use the same imaging biomarkers, tracers, or scan protocols in clinical ILD.

## 2. Experimental Section

### 2.1. Search Strategy and Search Protocol

This systematic review was performed in agreement with the PRISMA statement [22]. We performed a comprehensive literature search for articles concerning four main categories to address our aim with this review article. The categories combined were (I) Animal models, (II) Imaging, (III) Disease, and (IV) Drugs. Articles particularly of our interest were in vivo studies using live imaging. The animal models should, in addition, express ILD or pathological features resembling clinical affliction, such as inflammation, vascular leak, or fibrosis. In parallel, the structure of Population-Intervention-Comparison-Outcome (PICO) was followed when applicable [23].

### 2.2. Information Sources and Search Terms

**Figure 1:**
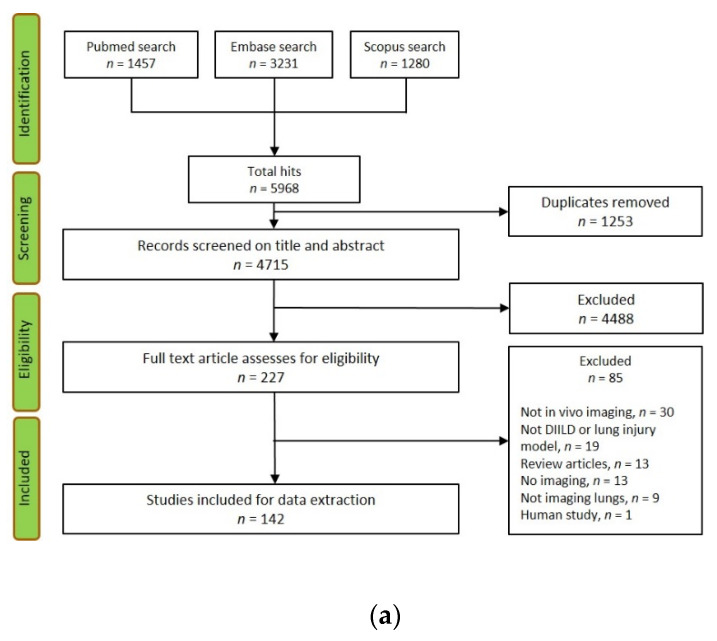

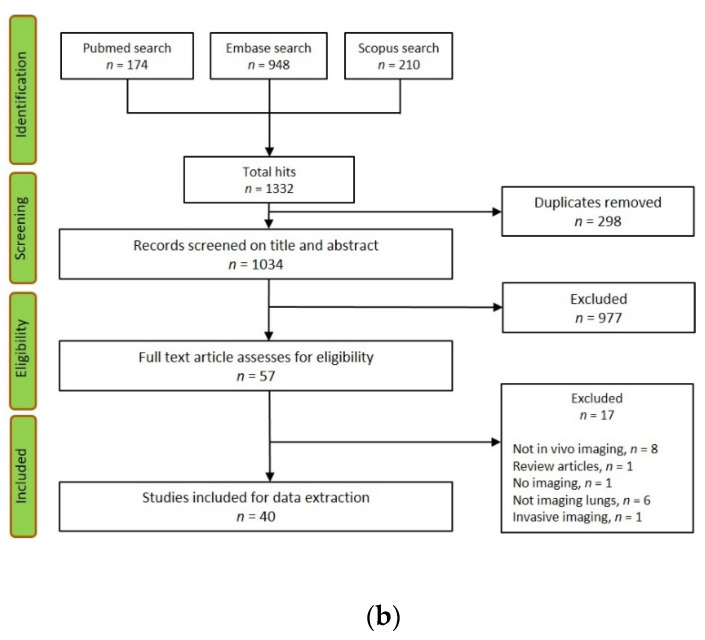
The flowchart of search hits and the different Preferred Reporting Items for Systematic reviews and Meta-analyses (PRISMA)-guideline selection phases, from the initial search (**a**) and the follow-up search (**b**), resulting in the total amount of 182 included articles.

Each of the main four search categories (Animal models, Imaging, Disease, and Drugs) were expanded into a list of search terms, listed in Supplementary data 1. The search was performed using the three databases PubMed, EMBASE, and Scopus, with combined search terms from all four categories. The search was limited to articles in press or published, reviews but not letter-to-the-editor or editorials, and we did not include so-called notes or conference abstracts. Other limitations were only articles written in English, articles that have an appurtenant abstract, and articles published from 1970. The initial search was performed to include publication years 1970–2017 (19th December 2017). A follow-up search was done thereafter, including articles from 2017–2019 (30th July 2019). The articles to be included from both searches needed to include imaging techniques, with imaging performed in living animals once or longitudinally, monitoring the lungs in particular. Subsequently, the three different databases were searched, and all hits were then merged, followed by removal of duplicates. The flow chart presented in Figure 1 shows the total number in each search process step. In addition, continuous search was performed within the category Drugs, reviewing the lung toxicology website www.pneumotox.com [6]. This was performed to possibly capture drug studies not yet published or newly reported drugs not investigated through clinical or preclinical studies to date.

### 2.3. Screening and Eligibility Process

After compiling all search hits from the three databases, duplicates were removed, and, subsequently, remaining articles were reviewed (screening process), based on the titles and abstracts. The eligibility of remaining articles was assessed based on the article full text version. Our initial search strategy did not exclude review articles, yet, at the eligibility stage, the authors decided not to extract data from articles that were so-called perspective articles or reviews. Only original articles were reviewed and underwent data extraction at the final selection stage. Inclusion and exclusion criteria, set beforehand by the authors, were defined and used as guidelines for the final selection process. In the supplementary material, in Supplementary data 2, the exclusion criteria are stated in Tables S1 and S2, alongside the number of articles listed for exclusion, together with the remaining description of the methods section.

## 3. Results & Highlights

### 3.1. Screening Assessment Based on Article Title and Abstract

From the first initial search, the total number of search hits from three databases was 5968 and after duplicate removal of 1253 articles, 4715 papers were surveyed by three reviewers, based on titles and abstracts (Figure 1A). From the screening process including 4715 papers, 227 (4.8%) articles were selected for full text evaluation. Among the 227 articles, additional 85 articles were excluded during the full text review, since the content did not match the inclusion criteria (Figure 1A). The total amount of 142 articles were finally included (3.0% of total hits), and data was extracted subsequently by nine reviewers.

From the follow-up search, 1332 articles were found, and after duplicate removal 1034 articles were kept for screening based on the title and the abstract. In total, 57 articles were further assessed as full text articles (5.5%) from the 1034 screened. The follow-up search resulted finally in additional 40 included articles from 57 evaluated ones, based on the full text version, as shown in (Figure 1B).

**Figure 2:**
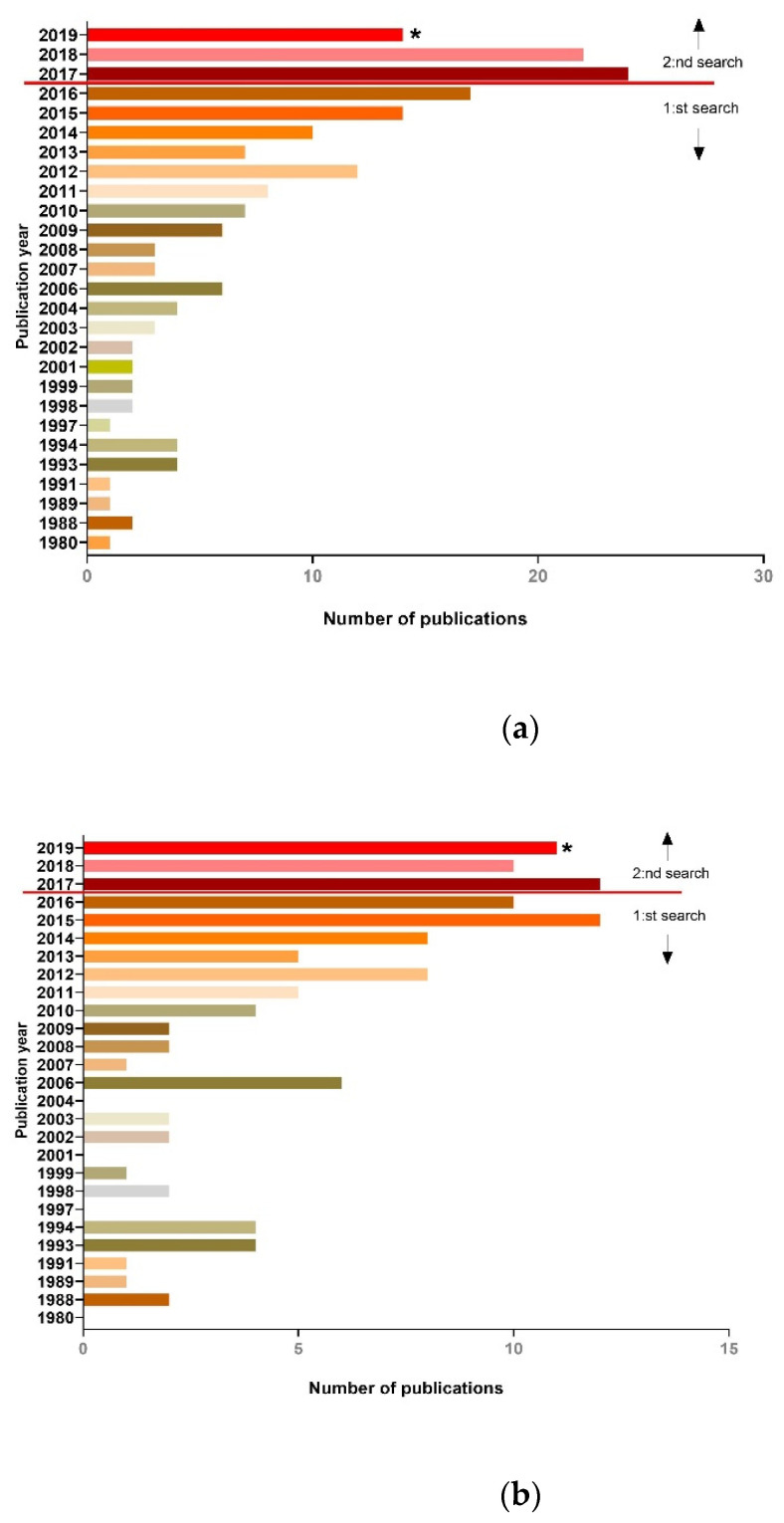
Time plots of the included articles that data was extracted from. Red line indicated articles that were included from the first and second search, respectively. (**a**) The total amount of articles published. (**b**) All articles that used longitudinal imaging as readout in their lung injury model. * = points out year 2019, which only includes articles published in the first half year of 2019 (January–July).

As the second search partly overlapped in time considering publication year 2017, several duplicates were detected among the first and second search. As the screening process was done by several reviewers, the exclusion of duplicates between the first and second search could not be guaranteed and, therefore, is presented separately as the first and a second follow-up search (Figure 1A,B). Another aspect of performing an updated search is to see how the statistic differences emerges when dealing with models and the advanced techniques in terms of live imaging. Clearly, live imaging in animal models is an increasingly used approach, and so is longitudinal monitoring, presented by the time distribution graphs (Figure 2).

All included articles are listed in a detailed reference list, in alphabetic order, in the Supplementary data 3. Within the total 182 included papers, only a few papers explicitly expressed that exploring DIILD was an aim of the study. However, the remaining papers were all lung injury models and included imaging techniques to study lung injury; thus, they were all highly relevant to include for research, with the focus of the review. All figures onward are presented as merged search results of the included papers with data extracted by three reviewers (I.M.P., K.v.W., and L.E.O.).

### 3.2. Animal Models

**Figure 3:**
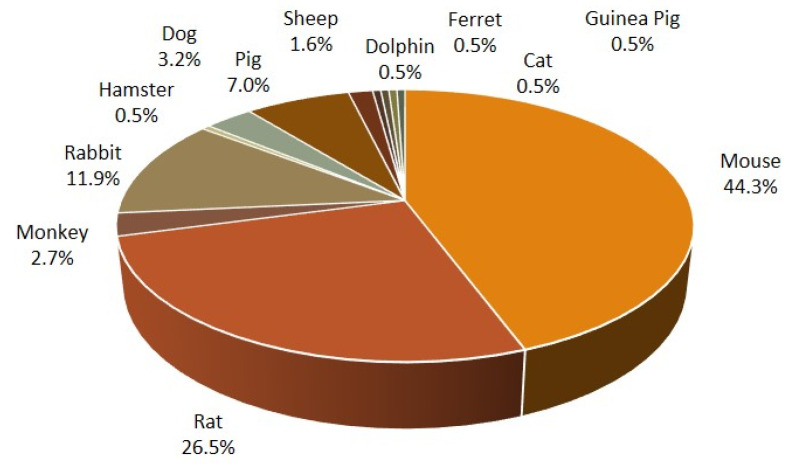
Animal species that were used for in vivo models to create lung injury. Mouse and rat models were the most abundantly used animals.

From the 182 articles, various models were carried out using 12 different animal species (Figure 3), where mouse was the largest group with more than 44% of all studies. Among the mouse studies, the C57BL/6 strain occurred in the majority (74%). Rat models were the second largest species, occurring in more than 26% of the studies, and were slightly more evenly distributed between strains compared to the mouse studies. The Sprague-Dawley strain was most abundant among the rat studies, with 50%, evident in Table 1. Rat studies are of particular interest in this context as most preclinical toxicology is performed in rats. Thirdly, rabbit models were found in nearly 12% of the selected studies, followed by pigs (7%) and dogs (3%). Other less studied animal species were hamster, ferret, and guinea pigs, but also larger animals, such as dolphins, sheep, and monkeys, occurred. One case study involving cats, was also included.

One important aspect of the design of an animal model is the lung injury agent and its route of administration. The agents used for induction of lung injury are listed in Table 2. The most frequently used lung injury inducing agents were bleomycin and lipopolysaccharide (LPS), with close to 34% and 10% of the selected studies, respectively. Irradiation was the third most common cause of lung injury for more than about 8% of the cases. The injury was induced by applying X-ray or high energy gamma radiation to the chest. Another common model to generate lung injury was inhalation of pure oxygen (6% of the models), which is based on damage triggered and created by reactive oxygen species (ROS). Infectious models (above 5%) and administration of oleic acid (in almost 6% of all models) were also used to induce lung injuries. Some studies used genetically modified animals (almost 5%, corresponding to 11 studies in total), where damage of the lung tissue was triggered or spontaneously developed over time. In addition, administration of elastase was used to produce experimental emphysema models (nearly 5% among all studies).

The route of administration of the inducing agents for lung injury methods are listed in Table 3. Intratracheal administration (i.t.) was the most frequently used administrative route, occurring in 41% of all studies. Inhalation or intravenous (i.v.) administration of the drug or injuring agent were applied for above 11% and at 9% of the 182 included studies, respectively.

From the 182 studies with lung injury models, almost 57% (a total of 103 studies) of them did not involve intervention or any type of drug treatment regime. In the 43% of the articles that included intervention groups, clear reversibility of lung injury could only be demonstrated in approximately 43% of these studies, as shown in Table 4. One example of an intervention study was where mesenchymal cells extracted from blood were injected i.v. to reduce LPS-induced lung injury [24]. Another example of treatment regime that was successful, was to use a somatostatin analogue to treat bleomycin-induced lung injury in rats [25]. The treatment decreased the gene expression of collagen type I and hydroxyproline levels in rat lungs, which are typical biomarkers of fibrosis. Newly emerged anti-fibrotic drugs nintedanib and pirfenidone in the clinical setting have proven to be successful for fibrosis treatment. In addition, one animal model applying one of these therapeutics was identified. Here, a bleomycin-induced lung injury was demonstrated using pirfenidone as a therapeutic approach, where lesions in the lungs of mice were assessed by PET-CT [26].

### 3.3. Aspects on Imaging Modalities, Techniques, and Tracers

**Figure 4:**
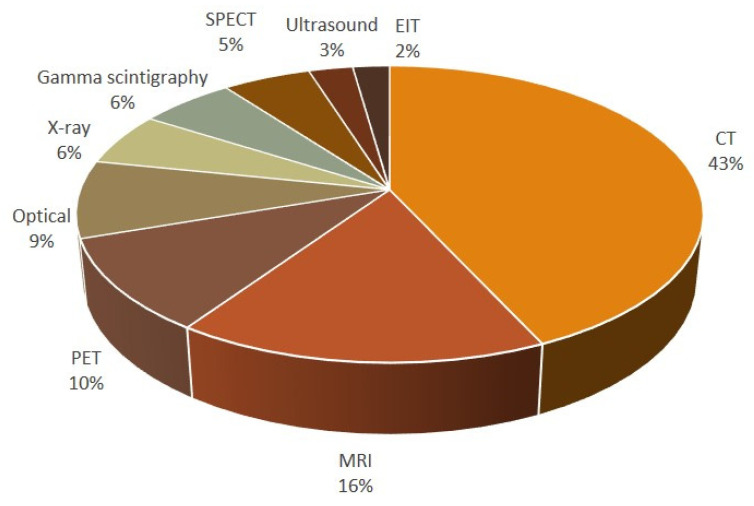
Imaging modalities used to monitor lung injury in vivo.

From the number of papers included, it is obvious that, for small animals such as rodents, dedicated devices are available for in vivo imaging. The imaging modality most frequently used among all 182 studies was CT (43%), followed by MRI (16%). Together with all the nuclear medicine techniques (PET, single-photon emission computed tomography (SPECT), gamma camera), these modalities represent 80% of the imaging techniques used (Figure 4).

For both CT and MRI, the signal from the lung tissue is in general low. Thereby, the two modalities can rather easily detect high-density inflammatory or fibrotic lesion against the dark background. However, the lesions can be hard to distinguish from vessels or other soft tissues present in the images. In terms of contrast, it should be noted that MRI has many advantages compared to CT for preclinical work, since MRI can offer many different endogenous contrast mechanisms. In addition, MRI has no exposure of ionizing radiation, which allows for longitudinal follow-up imaging without consideration of any accumulated radiation dose [27]. CT is the modality with highest spatial resolution (typical 0.04 mm in mouse and 0.1 mm in rat). MRI has, in general, somewhat lower spatial resolution (typical 0.1–0.2 mm in mouse and 0.2–0.3 mm in rat), but MRI can often compensate for the lower resolution by increased contrast. The nuclear medicine techniques have low inherent spatial resolution (typical > 0.4 mm) but can be highly specific due to the use of tracers, which monitor selected physiological processes [28,29].

### 3.4. Use of Radionuclide Tracers for Imaging

The literature of nuclear medicine studies shows various tracers and probes of choice, depending on the feasibility and scope of the study. PET tracers are dominated by ^18^F-fludeoxyglucose (FDG) with 39% occurrence among PET tracers, as well as in nearly 17% of all studies included in this review that applied radionuclides. This is the most common tracer in clinical use as well, and is mainly used for studies of glucose uptake and metabolism. It has been noted in clinical studies that there is a clear uptake of ^18^F-FDG in IPF patients [15]. However, it is unclear to what extent the uptake represents inflammatory or fibrotic processes. From animal studies using the bleomycin-induced lung injury, it has been shown that there is mainly ^18^F-FDG uptake in the lungs during the inflammatory phase. Small amounts of ^18^F-FDG uptake have been observed during the fibrotic phase, when there were no significant inflammatory processes on-going [30,31]. Since ^18^F-FDG cannot distinguish between the inflammatory and fibrotic processes, there is an urge for specific fibrotic tracers. Fibrosis can be characterized by excess deposition of collagens, primarily type I collagen [32]. Recently, a tracer specific for type I collagen was developed, ^68^Ga-CBP8, where a small peptide is conjugated to ^68^Ga that binds to newly synthesized collagen type I [33,34]. The tracer was firstly validated in a bleomycin mouse model using ^68^Ga PET-CT and then taken further into human studies [35]. Similar uptake of ^68^Ga-CBP8 was found by ex vivo analysis of lung tissue from patients with IPF [34]. Most recently, this Collagen-I binding peptide was also evaluated in a rat model, using the radionuclide ^64^Cu coupled to the small peptide CBP, for detection of newly synthesized Collagen-I in lung injury [36]. Among the PET studies, radiolabeled water (H_2_O^15^) or oxygen (^15^O) were also used for lung monitoring (14%). H_2_O^15^ and ^15^O are usually applied for ventilation and vascular leak imaging.

For the non-PET studies; ^99m^Tc and ^111^In are the dominating radionuclides. For ^99m^Tc, it is either used as pertechnetate or albumin, mainly for lung and vascular visualization. Increased pulmonary uptake of ^99m^Tc-hexamethylene-propylene amine oxime (HMPAO) has been observed for lung injury [37]. ^99m^Tc-HMPAO has been used to study DIILD and monitor the toxic effects of amiodarone therapy in a rabbit model [38]. ^111^In is the preferred radionuclide for labeling endogenous cells, typically neutrophils and antibodies. All tracers used for radionuclide techniques from the selected articles, and exemplified here in this review, are listed in Table 5.

### 3.5. MRI Contrast Agents

MRI can easily be combined with contrast agents. The deposition and clearance of gadopentetate aerosol has been used to monitor the lung injury from bleomycin [39]. However, the method was not well suited for fibrosis monitoring. A rapid clearance was found during the inflammatory phase, while the rate was back to normal during the fibrotic phase. Inhaled hyperpolarized gases can also be used as contrast agents for MRI. Lung injury induced by bleomycin has been studied with the inert gas helium-3, ^3^He [40]. This technique provided detailed information on ventilation and alveolar structure but no information regarding underlying pathophysiology. Lesions are depicted as signal voids only. MRI could also be used with other hyperpolarized gases, such as xenon-129, ^129^Xe. Xenon gas diffuses from the alveolar space into the blood and gas-exchanging tissues. This process offers several unique read-outs, which can benefit studies of lung injuries. Especially, the thickening and efficacy of the pulmonary blood-gas barrier can be measured. The method has proven to be sensitive to diffuse impairment caused by fibrotic thickening in a rat model with bleomycin [41]. Another study with LPS-induced lung injury indicated the benefit of using ^129^Xe where pulmonary diffusing capacity and perfusion was studied. The most evident readout was the total diffusion length being significantly changed in LPS-challenged animals compared to controls. In addition, capillary diffusion length was clearly augmented and detected by this imaging method [42]. Similar to ^3^He, ^129^Xe can also measure ventilation. MRI of hyperpolarized gases can be translated to patients. However, there is less experience from xenon imaging of lung injury in the clinic. It is worth to underline that the multitude of read-outs from xenon measurements will be extremely difficult to interpret in patients with lung injuries comprising a complex mixture of inflammatory and fibrotic processes.

### 3.6. Optical Imaging

Optical imaging is another imaging modality applied in 8% among all selected studies. As with the nuclear medicine methods, optical imaging can be highly specific due to the optical probe used for the experiment. Optical methods are nevertheless hampered by the rapid attenuation of light in tissue, but they work well in small animals, especially mice.

### 3.7. Duration of In Vivo Models and Longitudinal Imaging

One of the advantages with in vivo imaging is the possibility to perform longitudinal studies (at least two time points), as long as the imaging technique does not involve any invasive procedure for respiratory control. All the major imaging modalities can be used for longitudinal studies with repeated imaging. From the 182 selected studies, the majority of them were longitudinal studies (117 in total, corresponding to nearly 64%), applying two or more scan sessions over time to monitor changes in their lung injury model. The amount of repeated imaging sessions varied from 2–10, as presented in Table 6. Duration of the models varied from a few days up to several weeks, depending on the scope of the study and the expected pathological changes. The most clinically relevant animal models, with pathologies resembling lung injury or DIILD, were most often used in longitudinal imaging studies [37,43,44,45,46]. These, in particular, bring up the importance of individual variation or therapeutic outcome in disease models. An additional aspect to highlight when implementing imaging in model validation is that incidence of induced disease may also differ or create subgroups of pathologies worth discussing, as well as enables selection of treatment groups when only animals are chosen for continuous intervention experiments, where disease successfully was induced. Here, imaging biomarkers are extremely valuable, without the need to terminate any groups for validation of disease incidence [27,47].

### 3.8. Imaging and Respiratory Motion (Ventilator vs. Free Breathing)

In pulmonary imaging, the motion from the respiration may be an issue and source to image artefacts. In the clinical setting, this is often solved by imaging during breath hold. For animals, different approaches can be taken to address the motion of the lungs. For many experiments with moderate spatial resolution, the image quality is sufficient even though the animals are freely breathing and no gating technique is used, as shown by Jin et al., in 2012, applying CT; or in the case of MRI, shown by Babin et al. in 2011 [48,49]. For PET, most examinations are performed without any respiratory precautions. In order to take advantage of the high resolution that CT can offer, respiratory gating is needed [50]. The gating can rely on an internal anatomical marker or an external device, such as a pneumatic pillow. Additional control of the animal breathing can be achieved by intubation connected to a ventilator. This is a somewhat invasive technique, and for longitudinal studies, special care needs to be provided to make sure the animal is unaffected by the intubation procedure. The most elaborate method to control the respiratory pattern of an animal is to use tracheostomy and a ventilator. However, this is an invasive method, and the animal can only be imaged for one session. In order to use MRI with hyperpolarized gases, which may require extended breath-hold, ventilator-controlled respiration with tracheostomy of the animal is needed. It is thus very important to consider the invasiveness of the imaging method when aiming to perform longitudinal and translational studies.

### 3.9. Imaging- and Pathology Correlation

Regardless of the imaging technique used, most imaging data correlated with other biomarkers analyzed, such as histological assessment or comparison with invasive lung function measurements at termination [37,44,50,51]. Histopathology is considered the gold standard method to monitor the pathological changes of the lungs created by the exposure to bleomycin or any other agent. For studies of lung injury, mainly hematoxylin & eosin (H&E) and Masson’s trichrome staining techniques are used. More than 80% of the studies included in this review performed histopathology to independently monitor the disease model and to interpret, explain, or verify the imaging findings. Other applied techniques besides imaging were used in complementary purpose or to confirm the obtained imaging data.

A large amount of the studies had one or several histological analyses included in the study, with H&E being the most common staining technique. All histological staining techniques are listed in Table 7. Different types of immunohistochemistry were present, although they are summarized in Table 7 as one category. In addition to-, or instead of histology, many other assays and analysis methods were applied, such as lung function tests, protein expression profiling, or determination of hydroxyproline content in the lung tissue (Table 7).

In studies presenting both imaging data and histopathological results, correlations between the two types of biomarkers could be made. In the majority of papers, a correlation between imaging biomarkers and other biomarker(s) was reported in 62% of 182 total imaging articles, as presented in Table 8. In studies with the highest level of correlation with other biomarkers, MRI (81%) and CT (65%) were applied as imaging modalities. In studies using ultrasound and X-ray, the percentage of correlating studies was only 50% and 38%, respectively. As these figures relate to many different biomarkers, the comparison may be skewed by the fact that the biomarker used for comparison may be more or less validated. It is, therefore, difficult to draw any conclusions as to if some imaging modalities are more predictive than others. For this reason, we also compared how the different imaging modalities correlate to the most frequently used staining evaluation by H&E. Again, MRI generated the best correlations with 96% of studies, while CT only correlated in 66% of the papers with H&E histology.

The histopathology is often performed on a group level. In a limited number of studies, the imaging results were actually compared to the direct matching slice using histopathology [49,50]. In the study by Babin et al., in 2011, MR-images acquired from mice at day 7, day 28, and day 70 after bleomycin exposure were compared to the corresponding histopathological slices [49]. The lesions in the images have matching anatomical regions in the histopathological slice corresponding to inflammatory cells (day 7), fibrotic areas with some inflammatory cells (day 28), and multifocal fibrosis (day 70). Notably, there was no difference in the appearance (shape or intensity) of the lesions in the MR-images on the underlying histopathological background [49]. Thus, the identification of IBs that can detect and differentiate between inflammatory lesions and fibrotic lesions remains an unmet need.

In the study by Lee and colleagues, a very close agreement between the findings in CT images and histopathological scores was found from matching slices of lungs, from mice exposed to bleomycin [44]. The radiographic scoring during both the inflammatory phase and fibrotic phase correlated with pathologic reading. CT imaging was performed both in vivo and post-mortem. The post-mortem CT images have significant higher resolution and better image quality. Thereby, the accuracy of the radiographic reading improved, and the correlation to histopathology results increased, when the subject could be kept completely immobilized. Several examples of studies that applied histological findings to complement or confirm the imaging data were performed using CT as imaging modality [44,45,50,52]. Other important biomarkers that have been used to strengthen the imaging data have had different focuses. One study used profiling assays analyzing cytokine regulation at various time points that indicated interleukin (IL)-1β, IL-17, and IL-2 to be of importance in a bleomycin-induced lung injury model. This is in accordance with what has been observed previously in IPF [46]. Likewise, hydroxyproline has been used as a biomarker of increased collagen content in several studies [30,33,34,51,53,54,55,56]. Other methods that have been considered in association with imaging data are lung function testing, total- and differential cell count from bronchoalveolar lavage (BAL) samples, or ratio of the wet/dry weight of lung tissue.

### 3.10. Explicit DIILD Studies

The articles included in this review were lung injury models that employed imaging techniques, and large focus has been set to the translational value of these studies. Many relevant studies were found to be performed in a translational approach, where relevant physiological therapeutic doses were evaluated and where the insult or challenge used for creating the lung injury, inflammation, or fibrotic lesions were highly relevant in comparison to the clinical pathologies. However, studies explicitly expressing the aim to study DIILD were only a few out of 182 articles in total. In these studies, the actual side effects on the lung, induced by a drug were studied. Studies that used DIILD-inducing agents were amiodarone-induced lung injury studies [38,43,57]. Also, tetracycline [58] and lipiodol-associated [59] lesions were studied in two separate studies. Tetracycline was given intrapleural to rabbits, and lesions were followed by serial ultrasonography monitoring and CT, while lipiodol-induced lesions were monitored by fluoroscopy in rats. Amiodarone was administered orally in two of the studies [38,43], while the drug was administrated by intraperitoneal (i.p.) injection in the third study [57]. In the amiodarone studies, clinically translatable doses and duration of drug exposure were applied. In addition, animal groups with high-dose exposure were included, as well as long-term administration of low doses. All amiodarone experiments were carried out on rabbits, and longitudinal gamma scintigraphy imaging was used as readout. The pathological findings in the lungs that were obvious at termination were, however, not fully detected by imaging during disease progression. In one of the three studies, gamma scintigraphy was applied already after two weeks of exposure to amiodarone, due to the fact that animals died from lung toxicity before planned termination.

A substantial number of the lung injury models (72 articles in total, or almost 40%) partly addressed explicit DIILD-related questions. In those studies, bleomycin was used to create inflammation followed by fibrosis. Thus, the bleomycin-studies intended to create an injury that is well known and characterized by many bleomycin studies previously published [60]. Although bleomycin is a regularly used cancer drug with a clinical high incidence of DIILD, it was not drug-related side-effects from bleomycin that were investigated. Instead, bleomycin was used as an agent to create lung injury, without consideration of DIILD aspects. Most often bleomycin was expressed to represent a tool to create a pure fibrosis model (about 55% of all bleomycin studies). Some of the bleomycin studies expressed a particular aim to develop IPF models (almost 17%) or focused on the inflammatory phase (about 6%) of the bleomycin-induced changes. The pathological patterns in all these models was yet highly relevant to represent the effects from an agent, which cause drug-related lung injuries. Despite its limitations, the bleomycin-induced lung injury model may be considered the clinically most relevant model of DIILD to date. After all, there are limitations to the classical bleomycin model, where the bleomycin is administrated i.t., thus giving rise to an acute and initially pronounced inflammatory phase before fibrotic processes are initiated. In addition, this model potentially resolves after a few weeks, which would suggest the need for development of a chronic model involving repeated dosing of bleomycin, to better mimic the slowly progressing fibrosis evident in human. Yet, the classical bleomycin model seems to be a good tool to use for mimicking different aspects of lung disease and is frequently acknowledged for its reproducibility and yet comparable resemblance of fibrosis and IPF in humans [20,21]. The most drug-injury related studies are summarized in Table 9, mentioning the pathology that was expressed. The corresponding imaging techniques that were used in these studies are also related to histopathological data, and the reference presenting that particular study is mentioned in association to this work.

### 3.11. DIILD Models and Imaging Techniques

**Figure 5:**
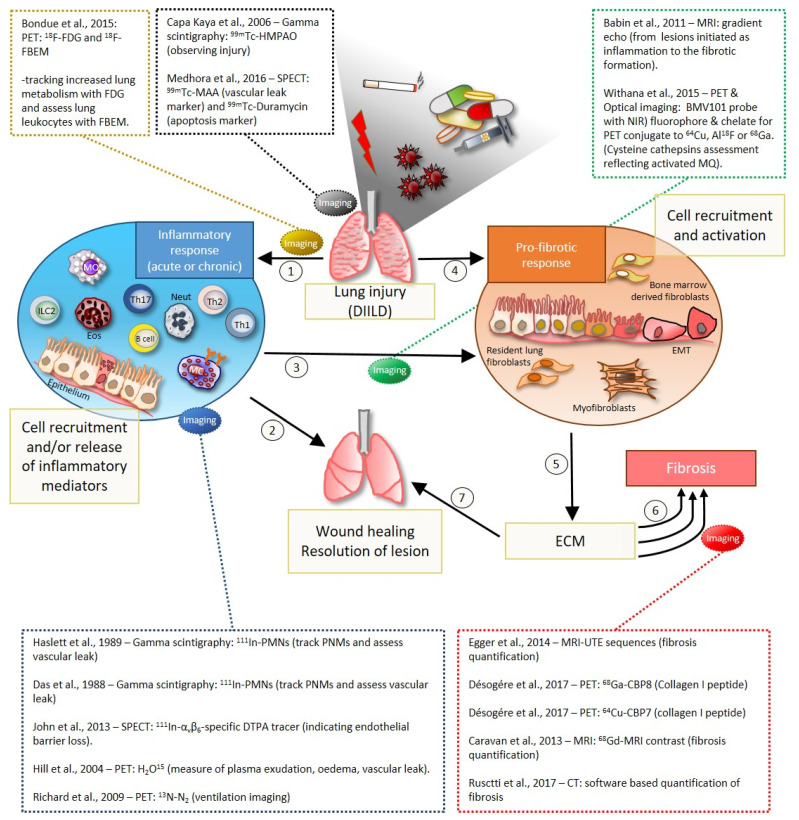
Summary of possible events occurring after drug-induced lung injury or other types of injury exposures, such as cigarette smoke, particle- or infectious agents, or simply mechanical injury. In association with the pathological scenarios that can occur, imaging techniques are used for detecting the lesions at various disease stages. (1) Drug-induced injury might lead to acute or chronic inflammation, involving immune cell infiltration and activation of the epithelium. This primes release of inflammatory mediators from activated epithelial cells and immune cell at the lung tissue injury site. (2) The inflammatory process is triggered in order to resolve and clear the injury, although the inflammation phase commonly leads to initiation of pro-fibrotic events (3). The fibrotic process might also occur directly after drug exposure, without necessarily the initial inflammation phase (4). The pro-fibrotic process might lead to epithelial mesenchymal transition (EMT). In addition, bone marrow derived fibroblasts might increase in the lung during the pro-fibrotic process, as well as resident lung fibroblasts that can transform into myofibroblasts, thus being able to release scar forming mediators and extracellular matrix (ECM) components (5). Elevated levels of ECM products might lead to fibrosis. And, once a stable scar is formed, the lung tissue loses the elastic property, giving rise to symptoms such as breathing difficulties and lung failure (6). If the initial fibrotic lesions can be resolved, then the lung damage can be limited and wound healing occurs in the lung (7). For each phase of DIILD, optimal imaging techniques can be used to assess the rise of lesions or monitor the inflammatory edematous lesion transforming into a fibrotic stable scar. Some of the methods using radionuclide or optical targeted imaging aim to track specific receptors, cells or metabolites, while computed tomography (CT) or magnetic resonance imaging (MRI) rather can be used to map the size and location of induced lesions in the lung.

In summary, imaging techniques can be used in a flexible manner and depending on the pathological changes or the setup of the disease model, different techniques are more or less suitable. In Figure 5, various phases of lung injury or DIILD are outlined. Drug-induced lung injury could develop with an initial inflammatory phase, being acute or chronic, but eventually lead to progression into fibrotic tissue when cells, such as fibroblasts and myofibroblasts, are activated [32,114]. The initiated production of extracellular matrix (ECM) components, such as collagens, for instance, are overproduced (Figure 5), and the fibrotic lesion may not resolve and remain as a stable scar in the lung tissue, or progress further into severe fibrosis until lung failure eventually occurs [32,114,115]. However, pro-fibrotic responses could also initiate ECM production, although contributing to wound healing and subsequently resolution of the lesion [114,116]. Based on the extracted data from the selected articles in this review, the summarizing Figure 5 indicates where different examples of imaging techniques are suggested, depending on which phase of the DIILD or lung injury that is of interest to study.

Suggested imaging techniques and tracers for inflammation monitoring were, for example, labeled polymorph-nuclear cells (PMN) monitoring neutrophil infiltration, or other strategies such as ventilation- or plasma leakage-monitoring in the lung, among several studies [63,92,117,118,119]. Studies with focus on the fibrosis detection or ECM production [25,33,34,50,55], among other markers or sequences, are also presented in Figure 5. Then, additionally suggested articles that were observing general aspects of lung injury [38,120] or tracking of disease progression when going from inflammation towards the fibrosis stage are presented in the figure [30,46,49].

## 4. Discussion

The aim of this systematic review was primarily to identify animal models of interstitial lung disease where non-invasive and in vivo imaging has been implemented. In addition, we investigated what type of imaging modalities have been used and to what extent potential IBs correlate with other biomarkers, such as histology. We sought to identify what imaging techniques that already had been reported in lung injury models and relevant DIILD models, and if any validated or preferred IBs were already available for the detection of lung injury. Applying IBs to assess ILD is a non-invasive technique. Imaging can also be suitable to monitor reversibility and can potentially be sensitive to focal changes. IBs can often be translatable between human and animal studies; therefore, it was of huge importance that the imaging was done in a non-invasive way and in live animals, according to the search criteria. Many valuable animal models of interstitial lung disease were found in this review with interesting imaging protocols, as given by a few examples in Figure 5. With improved IBs applied in lung injury models, the output of drug development and disease monitoring could possibly improve. Our literature search does not only identify the most important imaging studies for assessing lung disease and pathologies that resemble clinical ILD. This article also revealed the small fraction of the total number of studies that actually do include in vivo imaging in such models.

Among all search hits and included papers, not more than a few studies actually intended to investigate interstitial lung disease caused by administration of drugs, and applying imaging techniques to assess the pathological state of the lung, during DIILD. These studies involved the drugs amiodarone, tetracycline, and lipiodol. The adverse effects of amiodarone were investigated by gamma scintigraphy [38,43,57]. Even though the studies were performed with focus on translational aspects and DIILD, there were unfortunately pathological changes that were not fully reflected in the imaging results. There was a small correlation between the tracer uptake on gamma scintigraphy images and histopathological analyses once the animals were terminated. In addition, in this model, animals died during the experiments due to extensive yet not clearly apparent, lung injury that progressed into lung failure. These three studies showed that imaging data underestimated the pathological changes in the lungs of animals exposed to amiodarone. Although, using physiological doses and administration route for drug exposure, the repeated long-term administration of amiodarone in all three studies was set to appropriate time points for chronic exposure, being translatable to the scenario occurring in patients that are prescribed amiodarone. Considering the total amount of all 182 included studies, there is a need of model development, as such. Preferably more focus should be given to the development of translational ILD models where in vivo imaging techniques are applied to successfully detect progression of lung disease. Our review underlines the need to develop new and better models to improve the understanding for ILD overall but also for understanding the use of IBs in DIILD. In addition to the above studies on DIILD induced by amiodarone, tetracycline, and lipiodol, we also identified 72 articles with the intention to study pathologies relevant to DIILD, induced by bleomycin. The pathological changes that were studied in this model are relevant to DIILD, and among pathologies such as inflammation, vascular leak, edema, fibrosis, and general lung injury, were demonstrated. These studies are highly relevant and are therefore of importance for the summary of relevant papers selected within this review.

The most frequently used animal species were mice and rats, and the most common administration route was i.t. instillation. The majority of studies that involved i.t. route for drug administration indicate that most of the models use the direct exposure applied locally. This is often not the case in clinical manifestations of DIILD, where the drug is mostly administered intravenously or orally, thus being systemically available. Hence, in a clinical setting, the drug exposure will most probably induce the lung damage via the vascular system, subsequently reaching the surrounding lung tissue. The administration route may thus be of importance to consider when designing and conducting new in vivo models to increase translatability to the clinical scenario [121].

Not surprisingly, we found that CT was the most frequently used imaging modality. The need to consider potential side effects from ionizing radiation for preclinical pulmonary studies has been debated [122]. It is indisputable that extremely high radiation doses can be given to animals with the intention to acquire images of high spatial resolution. However, recently it was shown that the concern for the radiation dose is of minor importance, even for longitudinal studies with repeated measurements [122]. Longitudinal imaging studies are increasing over time (Figure 4). This is an important aspect in the clinical applications of IBs, thus being able to monitor patients throughout a therapeutic treatment of anti-inflammatory or anti-fibrotic treatment regime.

For pulmonary imaging, the major imaging modalities, namely CT, MRI, PET, and gamma camera examinations, can be translated with respect to the imaging technique, from preclinical experiments to a clinical setting, or vice versa. For MRI, the exact pulse sequences may not be available or suitable on both preclinical and clinical systems; however, MRI offers many degrees of freedom and in most cases a similar approach in forms of settings or sequences can be found. Imaging protocols where the patient holds their breath during image acquisition needs to be adjusted for animal use. One restriction with respect to translatability of imaging is the use of contrast agents and tracers. In preclinical research, agents or tracers may be employed which are not yet approved for human use. This is a limitation, and if the transfer of imaging techniques between the preclinical and clinical arena is the goal, non-approved agents or tracers should be avoided.

The results of this review clearly demonstrate the few available ILD studies with non-invasive imaging and a translational approach in model development, although this number is increasing each year as the imaging techniques are being improved and more available. There is yet an unmet need for lung injury-related models, as well as for translational IBs bridging between preclinical research and clinical use. As discussed above, the most frequently used animal model was the bleomycin-induced lung injury. This model has two distinct phases, the first being characterized by acute inflammation manifested by edema, followed by a second phase of progressive fibrosis. In addition, a third phase has been observed, described as the resolution phase, as both inflammation and fibrosis spontaneously regress with time in this model. Although, others studies suggest that the bleomycin model may not resolve completely, and this phenomenon might be affected by the age of the animals or simply by route of administration or dose given in the study [60,123,124,125]. The most frequently used imaging modality in the bleomycin model is CT, which measures density (or more precisely electron density). CT can monitor lesions associated with lung injuries, which have considerably higher density than lung parenchyma. Thus, CT cannot distinguish between edematous lesions and fibrosis with respect to density. Similarly, the signal measured by MRI detects lesions from the lung parenchyma by density differences. The generation of the MRI signal is much more complex than for CT. For example, by changing pulse sequence or echo times, different aspects of the parenchyma or lesions can be augmented. By calculating the relaxation times, additional information about the tissue can be obtained. Despite these additional possibilities, no MRI method is presently available that can distinguish edematous lesions and fibrosis, without using exogenous contrast media. For both CT and MRI, the read-out is most often the volume of the lesions. When the density in CT is expressed in Hounsfield Units (HU), the lesions can be objectively segmented from lung tissue. However, it is often difficult to distinguish lesions from soft tissue. Histogram analysis is one method to address this issue. In addition, CT images can be evaluated by radiographic scoring (ground-glass opacity, honeycomb, and other indicators), but the demand on the spatial resolutions for radiographic reading increases considerably. High spatial resolution may require gating or intubation in combination with mechanical ventilation. Thereby, the invasiveness of the experiment increases, as well as the radiation exposure. One obvious advantage of radiographic read-out of preclinical CT images is the translational aspect to the patient setting for which radiographic read-out is standard. In summary, both inflammatory and fibrotic lesions are visible by CT, as well as MRI; however, none of the in vivo imaging modalities alone can clearly distinguish the different phases of disease in the bleomycin model. At present, it is only specific PET tracers for collagen [33,34], which can distinguish fibrosis from inflammation. Since PET examinations are most often performed on a combined PET/CT equipment, a PET/CT examination has the potential to depict and distinguish the different aspects of the bleomycin model. The same capability would be valid for another less common hybrid imaging modality, i.e., PET/MRI. However, if the time point for imaging matches a phase of the model, which is clearly either inflammation or fibrosis, CT or MRI can be used as single modality. Histopathology and imaging data have been correlated or simply compared in the majority of the studies included in this review. However, among these observed studies that correlated histology with imaging data, MRI was found to be the imaging technique that best correlated between histological analyses and imaging readout, whereas SPECT and PET/CT correlated less well with histology. Since it is difficult to distinguish edema and inflammation from fibrosis by CT and MRI, it is evident that the correlations above must have been made only with respect to inflammation or fibrosis status versus normal healthy lung. In a recent study, comparison over time was done during inflammation and fibrosis in a 28-day bleomycin model in rats, by using a multi-imaging approach. Combined MRI and PET-FDG indicated however large heterogeneity of the lesions found in the lungs. Once the animals were separated by the severity of the lung injury according to pathological increment of the total lung volume, the difference in lesion volume of fibrotic versus inflammatory lesions were then partly seen by altering different echo-times by MRI, which was also confirmed by PET signal-uptake [126].

Histopathological data is the gold standard technique for evaluation of lung disease. Histopathology can be complemented with lung function measurements and gene- or protein analyses to confirm specific aspects of the disease at a certain time point or dosing regimen. The majority of the 182 papers that were assessed included one or multiple techniques, such as histology or gene profiling assays from lung tissue or BAL, to confirm their findings. In fact, more than 62% of the studies could correlate or strengthen the imaging data by other techniques. Most of these techniques were however invasive or required termination of the animals. Furthermore, almost two-thirds of the studies used their collected imaging data to present quantitative results, while others only showed arbitrary representation, scoring scales, or no quantitative data at all, but instead presented the results as representative images. In general, image analysis and quantitative methods are time consuming and demand knowledge. Therefore, along with warranted new lung injury models and translational IBs to develop, the quantitative methods also need to be optimized with increased accessibility and with protocols that are easy to perform.

Finally, it has become evident that imaging is being employed more frequently as a tool to study lung injury and points towards an exponentially increasing occurrence of the various techniques being used over the last decade. Probably, the most recent development of µCT has contributed to the large increase of the overall CT technique. Nevertheless, more radiotracers are also being developed and so are optical probes for specific immunological or pathological targeting in lung injury models, especially in small animal imaging.

One major limitation of the review is the search strategy and the combined search using all four different categories. By combining the search terms for studies with animal models of lung injury, in combination with imaging, the total outcome on number of articles from the search is limited. If the search was performed using a combination of only papers relevant for in vivo studies, or only imaging of the lung, respectively, then it would have resulted in higher number of total included studies with relevant lung imaging settings. However, the combination of imaging and ILD relevant model would be lacking. Another aspect to consider is that DIILD in particular has been reported more frequently in Japan, compared to rest of the world [2,3,10,127], generating non-English articles being excluded. Therefore, the scope of this review may be limited by the language. A strength of this review is that the search process has been performed in collaboration with a senior librarian with prior experience from creating search strategies for systematic reviews. In addition, the authors of this paper have long-standing experience from lung diseases, respiratory animal models, preclinical and clinical imaging, and drug development, including safety studies.

## 5. Conclusions

Several hundreds of drugs are known to cause DIILD and an increasing number of new drugs are regularly reported to cause respiratory problems [2,3]. In addition, lung injury and ILD overall are under-researched areas and are in need of good biomarkers for monitoring. Non-invasive biomarkers, such as IBs, would help to identify and characterize the incidence, but also the progression of ILD. Our findings indicate that there are various lung-injury models, although very few of them had an explicit scope to study DIILD. Several different imaging modalities were used, in the total 182 reviewed articles, monitoring lung injury in vivo. Here, we provide a better overview and knowledge of the imaging techniques available and how they can be used in lung injury imaging. Moreover, this review summarizes the most useful molecular imaging tracers and outlines their potential for functional readout in a translational manner. Optimally, it would be of great interest for future development of early biomarkers that could predict the disease progression in ILD. Furthermore, IBs often translate well between pre-clinical and clinical practice. Therefore, there is a strong need for new methods and IB to better evaluate and monitor ILD in patients.

## Figures and Tables

**Table 1:** The most frequently used strains from the mouse and rat studies.

Mouse Strain	Number of Studies	Percentage (%)
C57BL/6	66	74.2
Balb/c	14	15.7
FVB/N	2	2.3
129S6/SvEvTac	1	1.1
B6-/-129S6	1	1.1
Outbred strain	1	1.1
FRA2-transgenic	1	1.1
ddY strain	1	1.1
Hairless SKH-1	1	1.1
C3H/HeN	1	1.1
Total	89	100
**Rat Strain**	**Number of Studies**	**Percentage (%)**
Sprague-Dawley	25	50.0
Wistar	13	26.0
Brown Norway	5	10.0
Fisher	4	8.0
Lewis	1	2.0
Long Evans	1	2.0
WAG/RijCmcr	1	2.0
Total	50	100

Background color: for easy to read.

**Table 2:** Lung injury inducing agents or origin.

Injury Agent or Source	Occurrence	Percentage (%)
Bleomycin	72	33.8
LPS	22	10.3
Irradiation	18	8.5
Oxygen	13	6.1
Infection	12	5.6
Oleic acid	12	5.6
Spontaneous	11	5.2
Elastase	10	4.7
Mechanical lung injury	7	3.3
Acid aspiration	5	2.4
Monocrotaline	4	1.9
Amiodarone	3	1.4
Silica	3	1.4
OVA	3	1.4
Cigarette smoke	2	0.9
White smoke exposure	2	0.9
Nanoparticles	2	0.9
Lipiodol	1	0.5
Tetracycline	1	0.5
Endotoxin	1	0.5
Asbestos	1	0.5
Bone marrow and spleen cells	1	0.5
Cyanide	1	0.5
NO_2_ inhalation	1	0.5
Blocking agent	1	0.5
Gastric acid	1	0.5
Ablation (high power microwaves)	1	0.5
Capsaicin	1	0.5
Vascular leak agent	1	0.5
Total	213	100

**Table 3:** Administration route of lung injury substance or source of injury-inducing methods.

Administration Rout	Occurrence	Percentage (%)
Intratracheal (i.t.)	82	41.0
Inhalation	23	11.5
Intra venous (i.v.)	18	9.0
Irradiation	18	9.0
Intranasal (i.n.)	12	6.0
Intralobular (i.l.)	10	5.0
Spontaneous	9	4.5
Intraperitoneal (i.p.)	7	3.5
Oropharyngeal	6	3.0
Endotracheal	3	1.5
Subcutaneous (s.c.)	3	1.5
Gavage	2	1.0
Transtracheal (t.t.)	1	0.5
Intra-atrium	1	0.5
Intrapleural	1	0.5
Bronchoalveolar lavage	1	0.5
External insult	1	0.5
Retro-pharyngeal	1	0.5
Unspecified	1	0.5
Total	200	100

**Table 4:** Intervention and readout of the results from selected articles. Number of articles that included intervention in their studies and how many of those that showed reversibility of the lung injury. In addition, readout and the format in which the imaging data was presented, expressed either as quantified levels in unites, scoring systems, arbitrary units, or only shown as representative images.

Intervention Study	Number of Articles	Percentage (%)
No	103	56.6
Yes	79	43.4
Total	182	100
**Reversibility of Disease**	**Number of Articles**	**Percentage (%)**
No	17	21.5
Partly	28	35.4
Yes	34	43.1
Total	79	100
**Readout and Imaging Data Presentation**	**Number of Articles**	**Percentage (%)**
Quantification using units or algorithms	114	62.6
Score system	13	7.1
Arbitrary units	26	14.3
No quantification, only images presented	29	15.9
Total	182	100

**Table 5:** Tracers summarized in one table, separated by the imaging techniques where they were measured.

Imaging Technique	Tracer	Occurrence	Percentage (%)
PET	^18^F-FDG	11	17
	^18^F-FBEM (-4-fluorobenzamido-Nethylamino-maleimide) -leukocyte tracer	1	2
	^18^F-ML8 (apoptosis marker)	2	3
	^15^O_2_	2	3
	^64^Cu-CBP8 (tracking newly synthesized Collagen I)	1	2
	^68^Ga-CBP8 (tracking newly synthesized Collagen I)	1	2
	^64^Cu-NJB2	1	2
	^18^F-FTHA (fatty acid uptake)	1	2
	^18^F-FEDAC (translocator protein)	1	2
	^11^C-R-PK11195 (Specific macrophage binding molecule)	1	2
	^44^Sc-PPB-03 (binding to albumin)	1	2
	^15^H_2_O	2	3
	^67^Ga-citrate	1	2
	^64^Cu/Al^18^F/^68^Ga-BMV101 (binding activated macrophages)	1	2
	^13^N-N_2_ (ventilation imaging)	1	2
SPECT	^99m^Tc-MAA (micro-aggregated albumin)	3	5
	^99m^Tc-NaTc04	1	2
	^99m^Tc-AnnexinV	3	5
	^99m^Tc-DTPA (avB6)	1	2
	^99m^Tc-EC2 (folate receptor)	1	2
	^99m^Tc-pertechnate	1	2
	^99m^Tc-Duramycin	1	2
	^111^In-nanoparticles	1	2
	^177^Lu-DOTA-NOC (binding to somostatin receptor 2)	1	2
	^177^Lu-DOTA-RGD (binding to avB3)	1	2
	^177^Lu-PPB-01 (binding to albumin)	1	2
Gamma	^99m^Tc-Duramycin	2	3
scintigraphy	^99m^Tc-Tobramycin	1	2
	^99m^Tc-HMPAO	4	6
	^99m^Tc-MAA (micro-aggregated albumin)	1	2
	^99m^Tc-DTPA	1	2
	^99m^Tc-HSA (human serum albumin)	1	2
	^111^In-RSA (rat serum albumin)	1	2
	^111^In-ICAM-1	2	3
	^111^In-IgG	3	5
	^111^In-PMNs	4	6
	^67^Ga-citrate	2	3
	Total	65	100

**Table 6:** Total number of imaging sessions performed within the same animal. Longitudinal imaging was considered being two or more imaging occasions, enabling comparison of two various time points from the same animal.

Longitudinal Imaging	Number of Articles	Percentage (%)
No	65	35.7
Yes	117	64.3
Total	182	100
**Number of Longitudinal Imaging Sessions**	**Number of Articles**	**Percentage (%)**
2	25	21.4
3	24	20.5
4	23	19.7
5	7	5.9
>5	38	32.5
Total	117	100

**Table 7:** Invasive or terminating analyses performed for conformational and complementary purpose to the imaging data. Occurrence indicated presence of analyzing method, where several techniques could be used within one article. The second part of the table presents the different staining techniques and how frequently they were employed. * indicate protein expression assays, including both enzyme-linked immunosorbent assay (ELISA)-based analyses and use of the Western blot technique.

Assays and Methods Performed in Addition to Imaging	Occurrence	Percentage (%)
BAL	47	14.9
No additional analysis besides imaging	42	13.3
Protein expression *	39	12.4
Hydroxyproline	30	9.5
Lung function	19	6.0
PCR	17	5.4
Blood gas	15	4.8
Tracer/signal distribution in organs/lung	12	3.8
Heart rate or ECG	11	3.5
Serum markers	11	3.5
Edema measures wet/dry-weight of lungs	10	3.2
Apoptosis assays	9	2.9
Blood pressure	7	2.2
FACS	7	2.2
WBC count	7	2.2
MPO	6	1.9
Breathing rate	6	1.9
Oxidative stress marker	4	1.3
Alveolar parenchyma (wall thickness)	4	1.3
Cardiac output	4	1.3
Diff quick	3	1.0
Giemsa stain of immune cells	2	0.6
Lung PO_2_	2	0.6
Oxygen consumption	1	0.3
Total	315	100
**Lung Tissue Staining Techniques Post Mortem**	**Occurrence**	**Percentage (%)**
H&E	109	40.5
NO additional histological analysis besides imaging	38	14.1
Masson’s Trichrome stain	37	13.8
Immunohistochemistry	26	9.7
Sirius Red	20	7.4
Unspecified what type of tissue staining	9	3.4
Van Gieson staining	6	2.2
TUNEL	5	1.9
PAS	5	1.9
Bioluminescence, immunofluorescence	4	1.5
Reticulin staining	4	1.5
Acid-Fichin-Orange-G (AFOG) staining	2	0.7
Verhoeff-reaction staining	1	0.4
Pentachrome staining	1	0.4
Prussian blue	1	0.4
Ziehl-Nielsen staining	1	0.4
Total	269	100

**Table 8:** Number of studies with imaging and histopathological correlation.

Imaging- Histopathology Correlations			
Imaging Technique	Occurrence of Imaging (Total)	Total Number of Studies with Correlation	Percentage (%)
MRI	37	30	81
CT	97	63	65
PET	23	14	61
SPECT	12	7	58
Optical	19	11	58
Gamma scintigraphy	13	7	54
Ultrasound	6	3	50
X-ray	13	5	38
Electrical Impedance Tomography	5	0	0
	225	140	-
**Comparing all Studies with H&E**			
**Imaging Technique**	**Occurrence of Imaging (total)**	**Total Number of Studies with Correlation**	**Percentage (%)**
MRI	23	22	96
CT	65	43	56
PET	12	6	50
SPECT	9	6	67
Optical	8	5	63
Gamma scintigraphy	8	6	75
Ultrasound	4	2	50
X-ray	8	5	63
Electrical Impedance Tomography	0	0	0
	137	95	-

**Table 9:** The most drug-induced interstitial lung disease (DIILD)-related studies and the pathologies that were studied.

DIILD Related Studies					
Lung Injury Inducing Agent	Pathology	Animal Species	Imaging Modality	Correlation Imaging to Pathology	Reference
Amiodarone	Inflammation	Rabbit	Gamma scintigraphy	P	Capa Kaya, 2001 [43]
	Inflammation	Rabbit	Gamma scintigraphy	P	Durmus-Altun, 2004 [57]
	Inflammation	Rabbit	Gamma scintigraphy	N	Kaya, 2006 [38]
Tetracycline	Lung injury	Rabbit	Ultrasound & CT	Y	Komissarov, 2015 [58]
Lipiodol	Lung injury	Rat	Optical Imaging	P	Kwon, 2011 [59]
Bleomycin	Fibrosis	Pig	CT	Y	Balazs, 1994 [61]
	Lung injury, ILD	Dog	MRI, CT	Y	Suga, 2003 [39]
	Cough	Guinea Pig	CT	Y	Guo Y., 2019 [62]
	Inflammation, Vascular leak	Rabbit	Gamma scintigraphy	Y	Haslett, 1989 [63]
	IPF	Rabbit	MRI	Y	Kersjes, 1999 [64]
	Lung injury	Rabbit	PET	Y	Jones, 1998 [65]
	ILD	Rabbit	PET	P	Jones, 1994 [66]
	Fibrosis, ILD	Rabbit	CT	N	Gunther, 2003 [67]
	Fibrosis, ILD	Rabbit	CT	P	Hirose, 1993 [68]
	Lung injury	Rabbit	CT	Y	Lynch, 1997 [69]
	Fibrosis, ILD	Rabbit	CT	Y	Nagatani, 2011 [70]
	Lung injury, Inflammation	Rabbit	CT	P	Sonoda, 2014 [71]
	Fibrosis, ILD	Rabbit	CT	Y	Watanabe, 2013 [72]
	Lung injury, Inflammation	Rat	Gamma scintigraphy	Y	Weiner, 1998 [73]
	Inflammation, Fibrosis, ILD	Rat	MRI	Y	Cleveland, 2014 [41]
	Inflammation, Fibrosis, ILD	Rat	MRI	Y	Babin, 2011 [49]
	Fibrosis, ILD	Rat	MRI	Y	Couch, 2016 [74]
	Fibrosis	Rat	MRI	P	Jacob, 2010 [75]
	Fibrosis	Rat	MRI	P	Karmouty-Quintana, 2007 [76]
	ILD	Rat	MRI	Y	Stephen, 2010 [40]
	Fibrosis, ILD	Rat	CT, PET	Y	Xiong Y., 2018 [77]
	Fibrosis, ILD	Rat	CT, PET	Y	Xiong Y., 2019 [78]
	Fibrosis, ILD	Rat	CT, SPECT	N	Chamorro, 2018 [79]
	Fibrosis, ILD	Rat	CT	Y	Hubner, 2008 [52]
	Lung injury, Fibrosis	Rat	CT	Y	Johnson, 2007 [80]
	IPF	Rat	CT	Y	Yu, 2017 [81]
	Fibrosis	Rat	CT	P	Yu, 2015 [82]
	Lung injury, ARDS	Rat	Optical	Y	Chagnon, 2010 [83]
	Fibrosis, ILD	Rat & Mouse	MRI	N	Egger C., 2015 [84]
	IPF	Rat & Mouse	MRI	Y	Egger C., 2014 [25]
	Lung injury	Rat & Mouse	MRI	Y	Egger C., 2013 [85]
	Lung injury	Mouse	MRI	Y	Babin, 2012 [86]
	Inflammation, Fibrosis, ILD	Mouse	MRI	Y	Caravan, 2013 [55]
	Lung injury	Mouse	MRI	Y	Egger C., 2017 [87]
	ILD, Vascular leak	Mouse	MRI	Y	Hodono, 2018 [88]
	Fibrosis	Mouse	MRI	Y	Shea S.B., 2017 [89]
	Inflammation, Fibrosis, ILD	Mouse	MRI	Y	Waghorn, 2017 [90]
	IPF	Mouse	MRI, CT	Y	Vande Velde, 2014 [56]
	IPF	Mouse	MRI, SPECT	Y	Tassali, 2016 [91]
	ILD	Mouse	SPECT, CT	Y	John, 2013 [92]
	ILD	Mouse	SPECT, CT	Y	Schniering J., 2019 [93]
	Fibrosis	Mouse	SPECT, CT	P	Schniering J., 2018 [94]
	Lung injury, Vascular leak	Mouse	SPECT	Y	Kelderhouse, 2015 [95]
	Fibrosis	Mouse	SPECT, PET, CT	P	Schniering J., 2018 [96]
	Fibrosis, ILD	Mouse	PET, CT	Y	Bondue, 2015 [30]
	Fibrosis, Vascular leak	Mouse	PET, CT	P	Desogere P., 2017 [33]
	IPF	Mouse	PET, CT	Y	Desogere P., 2017 [34]
	Fibrosis	Mouse	PET, CT	N	Bondue, 2019 [26]
	Fibrosis	Mouse	PET, CT	Y	Jailkhani, 2019 [97]
	Fibrosis	Mouse	PET, CT, Optical imaging	P	Withana, 2016 [46]
	IPF, Fibrosis	Mouse	CT, Optical imaging	Y	Ruscitti, 2018 [98]
	Fibrosis	Mouse	Optical	N	Kelderhouse, 2016 [99]
	Fibrosis	Mouse	Optical	Y	Cai, 2013 [100]
	IPF	Mouse	Optical	Y	Stellari, 2017 [101]
	Inflammation, Fibrosis, ILD	Mouse	Optical	Y	LI, 2015 [102]
	Lung injury	Mouse	CT	Y	Ackermann, 2017 [51]
	Lung injury, Fibrosis	Mouse	CT	Y	Buonfiglio, 2016 [103]
	Fibrosis	Mouse	CT	Y	Cavanaugh, 2006 [104]
	Fibrosis	Mouse	CT	Y	Choi, 2014 [105]
	Fibrosis	Mouse	CT	Y	de Langhe, 2012 [53]
	IPF	Mouse	CT	Y	Jin, 2012 [48]
	ILD	Mouse	CT	P	Kimura, 2015 [106]
	IPF	Mouse	CT	Y	Lee, 2008 [44]
	Fibrosis	Mouse	CT	Y	Ruscitti, 2017 [50]
	IPF	Mouse	CT	N	Shofer, 2007 [107]
	Fibrosis	Mouse	CT	P	Shofer, 2008 [45]
	ILD	Mouse	CT	P	Vande Velde, 2016 [108]
	Fibrosis	Mouse	CT	Y	Fu, 2017 [109]
	Fibrosis	Mouse	CT	Y	Shao, 2019 [110]
	IPF	Mouse	CT	Y	Arora, 2018 [111]
	IPF	Mouse	CT	Y	Povedano, 2018 [112]
	Fibrosis	Mouse	X-ray	N	Hellbach, 2017 [113]

ILD, intersititial lung disease; IPF, idiopathic pulmonary fibrosis; CT, computed tomography; MRI, magnetic resonance imaging; PET, positron emission tomography; SPECT, single-photon emission computed tomography; P, partly; Y, yes; N, no.

## Data Availability

Data is contained within the article or as Supplementary Material.

## Supplementary Materials

The following are available online at https://www.mdpi.com/2077-0383/10/1/107/s1, Supplementary data 1: The four search categories with corresponding search terms. Supplementary data 2: Remaining description of the Material and Methods section with two tables; Table S1 summarizes the “Headings for data extraction”, and Table S2 demonstrates the eligibility criteria for excluded articles. Supplementary data 3: The 182 selected articles from which data was extracted and presented in this review. The references are listed in alphabetic order.

## Abbreviations

BAL	Broncho-alveolar lavage
BOOP	bronchiolitis obliterans organizing pneumonia
CT	computed tomography
DAD	diffuse alveolar damage
DIILD	drug induced interstitial lung disease
DTPA	diethylene-triamine-pentaacetate
HMPAO	hexamethylene-propylene amine oxime
IBs	imaging biomarkers
IMI	Innovative Medicines Initiative
IPF	idiopathic pulmonary fibrosis
i.t.	intratracheal
i.v.	intravenous
i.l.	intralobular
i.n.	intranasal
i.p.	intraperitoneal
LPS	lipopolysaccharide
MRI	magnetic resonance imaging
NSIP	nonspecific interstitial pneumonia
TRISTAN	Translational Imaging in Drug Safety Assessment
PET	positron emission tomography
PRISMA	Preferred Reporting Items for Systematic reviews and Meta-analyses
SPECT	Single-photon emission computed tomography
s.c.	subcutaneous
TNF-α	Tumor necrosis factor-alpha
t.t.	transtracheal
UIP	usual interstitial pneumonia-like pattern

## References

[1] Skeoch S., Weatherley N., Swift A.J., Oldroyd A., Johns C., Hayton C., Giollo A., Wild J.M., Waterton J.C., Buch M. (2018). Drug-Induced Interstitial Lung Disease: A Systematic Review. J. Clin. Med..

[2] Schwaiblmair M., Behr W., Haeckel T., Markl B., Foerg W., Berghaus T. (2012). Drug induced interstitial lung disease. Open Respir. Med. J..

[3] Matsuno O. (2012). Drug-induced interstitial lung disease: Mechanisms and best diagnostic approaches. Respir. Res..

[4] Delaunay M., Cadranel J., Lusque A., Meyer N., Gounant V., Moro-Sibilot D., Michot J.M., Raimbourg J., Girard N., Guisier F. (2017). Immune-checkpoint inhibitors associated with interstitial lung disease in cancer patients. Eur. Respir. J..

[5] Flieder D.B., Travis W.D. (2004). Pathologic characteristics of drug-induced lung disease. Clin. Chest Med..

[6] Drug-Toxicity Tracking Webpage Pneumotox. https://www.pneumotox.com/drug/index/:2018.

[7] Travis W.D., Costabel U., Hansell D.M., King T.E., Lynch D.A., Nicholson A.G., Ryerson C.J., Ryu J.H., Selman M., Wells A.U. (2013). An official American Thoracic Society/European Respiratory Society statement: Update of the international multidisciplinary classification of the idiopathic interstitial pneumonias. Am. J. Respir. Crit. Care Med..

[8] Antoniou K.M., Margaritopoulos G.A., Tomassetti S., Bonella F., Costabel U., Poletti V. (2014). Interstitial lung disease. Eur. Respir. Rev..

[9] Eaden J., Chan H.-F., Hughes P., Weatherly N., Austin M., Smith L., Lithgow J., Swift A., Renshaw S., Buch M. (2019). Hyperpolarised 129-xenon diffusion-weighted MRI in interstitial lung disease. Eur. Respir. J..

[10] Saito Y., Gemma A. (2012). Current status of DILD in molecular targeted therapies. Int. J. Clin. Oncol..

[11] Rossi S.E., Erasmus J.J., McAdams H.P., Sporn T.A., Goodman P.C. (2000). Pulmonary drug toxicity: Radiologic and pathologic manifestations. Radiographics.

[12] Cavagna L., Monti S., Grosso V., Boffini N., Scorletti E., Crepaldi G., Caporali R. (2013). The multifaceted aspects of interstitial lung disease in rheumatoid arthritis. Biomed. Res. Int..

[13] Weatherley N.D., Eaden J.A., Stewart N.J., Bartholmai B.J., Swift A.J., Bianchi S.M., Wild J.M. (2019). Experimental and quantitative imaging techniques in interstitial lung disease. Thorax.

[14] Ebner L., Kammerman J., Driehuys B., Schiebler M.L., Cadman R.V., Fain S.B. (2017). The role of hyperpolarized (129)xenon in MR imaging of pulmonary function. Eur. J. Radiol..

[15] Justet A., Laurent-Bellue A., Thabut G., Dieudonne A., Debray M.P., Borie R., Aubier M., Lebtahi R., Crestani B. (2017). [(18)F]FDG PET/CT predicts progression-free survival in patients with idiopathic pulmonary fibrosis. Respir. Res..

[16] Win T., Lambrou T., Hutton B.F., Kayani I., Screaton N.J., Porter J.C., Maher T.M., Endozo R., Shortman R.I., Lukey P. (2012). 18F-Fluorodeoxyglucose positron emission tomography pulmonary imaging in idiopathic pulmonary fibrosis is reproducible: Implications for future clinical trials. Eur. J. Nucl. Med. Mol. Imaging.

[17] Mammarappallil J.G., Rankine L., Wild J.M., Driehuys B. (2019). New Developments in Imaging Idiopathic Pulmonary Fibrosis with Hyperpolarized Xenon Magnetic Resonance Imaging. J. Thorac. Imaging.

[18] Kubo K., Azuma A., Kanazawa M., Kameda H., Kusumoto M., Genma A., Saijo Y., Sakai F., Sugiyama Y., Tatsumi K. (2013). Consensus statement for the diagnosis and treatment of drug-induced lung injuries. Respir. Investig..

[19] TRISTAN Consortium. https://www.imi-tristan.eu.

[20] Bonniaud P., Fabre A., Frossard N., Guignabert C., Inman M., Kuebler W.M., Maes T., Shi W., Stampfli M., Uhlig S. (2018). Optimising experimental research in respiratory diseases: An ERS statement. Eur. Respir. J..

[21] Jenkins R.G., Moore B.B., Chambers R.C., Eickelberg O., Konigshoff M., Kolb M., Laurent G.J., Nanthakumar C.B., Olman M.A., Pardo A. (2017). An Official American Thoracic Society Workshop Report: Use of Animal Models for the Preclinical Assessment of Potential Therapies for Pulmonary Fibrosis. Am. J. Respir. Cell Mol. Biol..

[22] Moher D., Liberati A., Tetzlaff J., Altman D.G., Group P. (2009). Preferred reporting items for systematic reviews and meta-analyses: The PRISMA Statement. Open Med..

[23] Schardt C., Adams M.B., Owens T., Keitz S., Fontelo P. (2007). Utilization of the PICO framework to improve searching PubMed for clinical questions. BMC Med. Inf. Decis. Mak..

[24] Xiang B., Chen L., Wang X., Zhao Y., Wang Y., Xiang C. (2017). Transplantation of Menstrual Blood-Derived Mesenchymal Stem Cells Promotes the Repair of LPS-Induced Acute Lung Injury. Int. J. Mol. Sci..

[25] Egger C., Gerard C., Vidotto N., Accart N., Cannet C., Dunbar A., Tigani B., Piaia A., Jarai G., Jarman E. (2014). Lung volume quantified by MRI reflects extracellular-matrix deposition and altered pulmonary function in bleomycin models of fibrosis: Effects of SOM230. Am. J. Physiol. Lung Cell. Mol. Physiol..

[26] Bondue B., Castiaux A., Van Simaeys G., Mathey C., Sherer F., Egrise D., Lacroix S., Huaux F., Doumont G., Goldman S. (2019). Absence of early metabolic response assessed by 18F-FDG PET/CT after initiation of antifibrotic drugs in IPF patients. Respir. Res..

[27] Barreto M.M., Rafful P.P., Rodrigues R.S., Zanetti G., Hochhegger B., Souza A.S., Guimaraes M.D., Marchiori E. (2013). Correlation between computed tomographic and magnetic resonance imaging findings of parenchymal lung diseases. Eur. J. Radiol..

[28] Young H.M., Eddy R.L., Parraga G. (2019). MRI and CT lung biomarkers: Towards an in vivo understanding of lung biomechanics. Clin. Biomech. (Bristol Avon).

[29] Bianchi A., Tibiletti M., Kjorstad A., Birk G., Schad L.R., Stierstorfer B., Rasche V., Stiller D. (2015). Three-dimensional accurate detection of lung emphysema in rats using ultra-short and zero echo time MRI. NMR Biomed..

[30] Bondue B., Sherer F., Van Simaeys G., Doumont G., Egrise D., Yakoub Y., Huaux F., Parmentier M., Rorive S., Sauvage S. (2015). PET/CT with 18F-FDG- and 18F-FBEM-labeled leukocytes for metabolic activity and leukocyte recruitment monitoring in a mouse model of pulmonary fibrosis. J. Nucl. Med..

[31] Scherer P.M., Chen D.L. (2016). Imaging Pulmonary Inflammation. J. Nucl. Med..

[32] Wynn T.A., Ramalingam T.R. (2012). Mechanisms of fibrosis: Therapeutic translation for fibrotic disease. Nat. Med..

[33] Desogere P., Tapias L.F., Rietz T.A., Rotile N., Blasi F., Day H., Elliott J., Fuchs B.C., Lanuti M., Caravan P. (2017). Optimization of a Collagen-Targeted PET Probe for Molecular Imaging of Pulmonary Fibrosis. J. Nucl. Med..

[34] Desogere P., Tapias L.F., Hariri L.P., Rotile N.J., Rietz T.A., Probst C.K., Blasi F., Day H., Mino-Kenudson M., Weinreb P. (2017). Type I collagen-targeted PET probe for pulmonary fibrosis detection and staging in preclinical models. Sci. Transl. Med..

[35] Montesi S.B., Izquierdo-Garcia D., Desogere P., Abston E., Liang L.L., Digumarthy S., Seethamraju R., Lanuti M., Caravan P., Catana C. (2019). Type I Collagen-targeted Positron Emission Tomography Imaging in Idiopathic Pulmonary Fibrosis: First-in-Human Studies. Am. J. Respir. Crit. Care Med..

[36] Mahmutovic Persson I., Fransén Pettersson N., Liu J., Falk Håkansson H., Örbom A., In ’t Zandt R., Gidlöf R., Sydoff M., von Wachenfeldt K., Olsson L.E. (2020). Longitudinal Imaging Using PET/CT with Collagen-I PET-Tracer and MRI for Assessment of Fi-brotic and Inflammatory Lesions in a Rat Lung Injury Model. J. Clin. Med..

[37] Suga K., Uchisako H., Nishigauchi K., Shimizu K., Kume N., Yamada N., Nakanishi T. (1994). Technetium-99m-HMPAO as a marker of chemical and irradiation lung injury: Experimental and clinical investigations. J. Nucl. Med..

[38] Kaya G.C., Ertay T., Tuna B., Bekis R., Tasci C., Sayit E., Yilmaz O., Kargi A., Durak H. (2006). Technetium-99m hexamethylpropylene amine oxime lung scintigraphy findings in low-dose amiodarone therapy. Lung.

[39] Suga K., Yuan Y., Ogasawara N., Tsukuda T., Matsunaga N. (2003). Altered clearance of gadolinium diethylenetriaminepentaacetic acid aerosol from bleomycin-injured dog lungs: Initial observations. Am. J. Respir. Crit. Care Med..

[40] Stephen M.J., Emami K., Woodburn J.M., Chia E., Kadlecek S., Zhu J., Pickup S., Ishii M., Rizi R.R., Rossman M. (2010). Quantitative assessment of lung ventilation and microstructure in an animal model of idiopathic pulmonary fibrosis using hyperpolarized gas MRI. Acad. Radiol..

[41] Cleveland Z.I., Virgincar R.S., Qi Y., Robertson S.H., Degan S., Driehuys B. (2014). 3D MRI of impaired hyperpolarized 129Xe uptake in a rat model of pulmonary fibrosis. NMR Biomed..

[42] Mansson S., Wolber J., Driehuys B., Wollmer P., Golman K. (2003). Characterization of diffusing capacity and perfusion of the rat lung in a lipopolysaccaride disease model using hyperpolarized 129Xe. Magn. Reson. Med..

[43] Capa Kaya G., Bekis R., Kirimca F., Ertay T., Kargi A., Gure A., Durak H. (2001). Use of technetium-99m HMPAO scintigraphy for the detection of amiodarone lung toxicity in a rabbit model. Eur. J. Nucl. Med..

[44] Lee H.J., Goo J.M., Kim N.R., Kim M.A., Chung D.H., Son K.R., Kim H.C., Lee C.H., Park C.M., Chun E.J. (2008). Semiquantitative measurement of murine bleomycin-induced lung fibrosis in in vivo and postmortem conditions using microcomputed tomography: Correlation with pathologic scores—initial results. Investig. Radiol..

[45] Shofer S., Badea C., Qi Y., Potts E., Foster W.M., Johnson G.A. (2008). A micro-CT analysis of murine lung recruitment in bleomycin-induced lung injury. J. Appl. Physiol. (1985).

[46] Withana N.P., Ma X., McGuire H.M., Verdoes M., van der Linden W.A., Ofori L.O., Zhang R., Li H., Sanman L.E., Wei K. (2016). Non-invasive Imaging of Idiopathic Pulmonary Fibrosis Using Cathepsin Protease Probes. Sci. Rep..

[47] Tielemans B., Dekoster K., Verleden S.E., Sawall S., Leszczynski B., Laperre K., Vanstapel A., Verschakelen J., Kachelriess M., Verbeken E. (2020). From Mouse to Man and Back: Closing the Correlation Gap between Imaging and Histopathology for Lung Diseases. Diagnostics (Basel).

[48] Jin G.Y., Bok S.M., Han Y.M., Chung M.J., Yoon K.H., Kim S.R., Lee Y.C. (2012). Effectiveness of rosiglitazone on bleomycin-induced lung fibrosis: Assessed by micro-computed tomography and pathologic scores. Eur. J. Radiol..

[49] Babin A.L., Cannet C., Gérard C., Wyss D., Page C.P., Beckmann N. (2011). Noninvasive assessment of bleomycin-induced lung injury and the effects of short-term glucocorticosteroid treatment in rats using MRI. J. Magn. Reson. Imaging.

[50] Ruscitti F., Ravanetti F., Essers J., Ridwan Y., Belenkov S., Vos W., Ferreira F., KleinJan A., Van Heijningen P., Van Holsbeke C. (2017). Longitudinal assessment of bleomycin-induced lung fibrosis by Micro-CT correlates with histological evaluation in mice. Multidiscip. Respir. Med..

[51] Ackermann M., Kim Y.O., Wagner W.L., Schuppan D., Valenzuela C.D., Mentzer S.J., Kreuz S., Stiller D., Wollin L., Konerding M.A. (2017). Effects of nintedanib on the microvascular architecture in a lung fibrosis model. Angiogenesis.

[52] Hubner R.H., Gitter W., El Mokhtari N.E., Mathiak M., Both M., Bolte H., Freitag-Wolf S., Bewig B. (2008). Standardized quantification of pulmonary fibrosis in histological samples. Biotechniques.

[53] De Langhe E., Vande Velde G., Hostens J., Himmelreich U., Nemery B., Luyten F.P., Vanoirbeek J., Lories R.J. (2012). Quantification of lung fibrosis and emphysema in mice using automated micro-computed tomography. PLoS ONE.

[54] Cleveland Z.I., Zhou Y.M., Akinyi T.G., Dunn R.S., Davidson C.R., Guo J., Woods J.C., Hardie W.D. (2017). Magnetic resonance imaging of disease progression and resolution in a transgenic mouse model of pulmonary fibrosis. Am. J. Physiol. Lung Cell. Mol. Physiol..

[55] Caravan P., Yang Y., Zachariah R., Schmitt A., Mino-Kenudson M., Chen H.H., Sosnovik D.E., Dai G., Fuchs B.C., Lanuti M. (2013). Molecular magnetic resonance imaging of pulmonary fibrosis in mice. Am. J. Respir. Cell Mol. Biol..

[56] Vande Velde G., De Langhe E., Poelmans J., Dresselaers T., Lories R.J., Himmelreich U. (2014). Magnetic resonance imaging for noninvasive assessment of lung fibrosis onset and progression: Cross-validation and comparison of different magnetic resonance imaging protocols with micro-computed tomography and histology in the bleomycin-induced mouse model. Investig. Radiol..

[57] Durmus-Altun G., Altun A., Sami Salihoglu Y., Altaner S., Berkada S., Ozbay G. (2004). Value of technetium-99m diethyltriamine pentaaceticacid radioaerosol inhalation lung scintigraphy for the stage of amiodarone-induced pulmonary toxicity. Int. J. Cardiol..

[58] Komissarov A.A., Florova G., Azghani A.O., Buchanan A., Bradley W.M., Schaefer C., Koenig K., Idell S. (2015). The time course of resolution of adhesions during fibrinolytic therapy in tetracycline-induced pleural injury in rabbits. Am. J. Physiol. Lung Cell. Mol. Physiol..

[59] Kwon W.J., Kim H.J., Jeong Y.J., Lee C.H., Kim K.I., Kim Y.D., Lee J.H. (2011). Direct lipiodol injection used for a radio-opaque lung marker: Stability and histopathologic effects. Exp. Lung Res..

[60] Della Latta V., Cecchettini A., Del Ry S., Morales M.A. (2015). Bleomycin in the setting of lung fibrosis induction: From biological mechanisms to counteractions. Pharm. Res..

[61] Balazs G., Noma S., Khan A., Eacobacci T., Herman P.G. (1994). Bleomycin-induced fibrosis in pigs: Evaluation with CT. Radiology.

[62] Guo Y., Ying S., Zhao X., Liu J., Wang Y. (2019). Increased expression of lung TRPV1/TRPA1 in a cough model of bleomycin-induced pulmonary fibrosis in Guinea pigs. BMC Pulm. Med..

[63] Haslett C., Shen A.S., Feldsien D.C., Allen D., Henson P.M., Cherniack R.M. (1989). 111Indium-labeled neutrophil migration into the lungs of bleomycin-treated rabbits assessed noninvasively by external scintigraphy. Am. Rev. Respir. Dis..

[64] Kersjes W., Hildebrandt G., Cagil H., Schunk K., Von Zitzewitz H., Schild H. (1999). Differentiation of alveolitis and pulmonary fibrosis in rabbits with magnetic resonance imaging after intrabronchial administration of bleomycin. Investig. Radiol..

[65] Jones H.A., Schofield J.B., Krausz T., Boobis A.R., Haslett C. (1998). Pulmonary fibrosis correlates with duration of tissue neutrophil activation. Am. J. Respir. Crit. Care Med..

[66] Jones H.A., Clark R.J., Rhodes C.G., Schofield J.B., Krausz T., Haslett C. (1994). In vivo measurement of neutrophil activity in experimental lung inflammation. Am. J. Respir. Crit. Care Med..

[67] Gunther A., Lubke N., Ermert M., Schermuly R.T., Weissmann N., Breithecker A., Markart P., Ruppert C., Quanz K., Ermert L. (2003). Prevention of bleomycin-induced lung fibrosis by aerosolization of heparin or urokinase in rabbits. Am. J. Respir. Crit. Care Med..

[68] Hirose N., Lynch D.A., Cherniack R.M., Doherty D.E. (1993). Correlation between high resolution computed tomography and tissue morphometry of the lung in bleomycin-induced pulmonary fibrosis in the rabbit. Am. Rev. Respir. Dis..

[69] Lynch D.A., Hirose N., Cherniack R.M., Doherty D.E. (1997). Bleomycin-induced lung disease in an animal model: Correlation between computed tomography-determined abnormalities and lung function. Acad. Radiol..

[70] Nagatani Y., Nitta N., Otani H., Mukaisho K., Sonoda A., Nitta-Seko A., Takahashi M., Murata K. (2011). Quantitative Measurement of Bleomycin-induced Lung Fibrosis in Rabbits Using Sequential in vivo Regional Analysis and High-Resolution Computed Tomography: Correlation with Pathologic Findings. Acad. Radiol..

[71] Sonoda A., Nitta N., Tsuchiya K., Otani H., Watanabe S., Mukaisho K., Tomozawa Y., Nagatani Y., Ohta S., Takahashi M. (2014). Asialoerythropoietin ameliorates bleomycin-induced acute lung injury in rabbits by reducing inflammation. Exp. Ther. Med..

[72] Watanabe S., Nitta N., Sonoda A., Nitta-Seko A., Ohta S., Tsuchiya K., Otani H., Tomozawa Y., Nagatani Y., Mukaisho K. (2013). Inhibition of fibrosis and inflammation by triple therapy with pirfenidone, edaravone and erythropoietin in rabbits with drug-induced lung injury: Comparison of CT imaging and pathological findings. Exp. Ther. Med..

[73] Weiner R.E., Sasso D.E., Gionfriddo M.A., Syrbu S.I., Smilowitz H.M., Vento J., Thrall R.S. (1998). Early detection of bleomycin-induced lung injury in rat using indium- 111-labeled antibody directed against intercellular adhesion molecule-1. J. Nucl. Med..

[74] Couch M.J., Fox M.S., Viel C., Gajawada G., Li T., Ouriadov A.V., Albert M.S. (2016). Fractional ventilation mapping using inert fluorinated gas MRI in rat models of inflammation and fibrosis. NMR Biomed..

[75] Jacob R.E., Amidan B.G., Soelberg J., Minard K.R. (2010). In vivo MRI of altered proton signal intensity and T2 relaxation in a bleomycin model of pulmonary inflammation and fibrosis. J. Magn. Reson. Imaging.

[76] Karmouty-Quintana H., Cannet C., Zurbruegg S., Blé F.X., Fozard J.R., Page C.P., Beckmann N. (2007). Bleomycin-induced lung injury assessed noninvasively and in spontaneously breathing rats by proton MRI. J. Magn. Reson. Imaging.

[77] Xiong Y., Nie D., Liu S., Ma H., Su S., Sun A., Zhao J., Zhang Z., Xiang X., Tang G. (2018). Apoptotic PET Imaging of Rat Pulmonary Fibrosis with [^18^F]ML-8. Crit. Care.

[78] Xiong Y., Nie D., Liu S., Ma H., Su S., Sun A., Zhao J., Zhang Z., Xiang X., Tang G. (2019). Apoptotic PET Imaging of Rat Pulmonary Fibrosis with Small-Molecule Radiotracer. Mol. Imaging Biol..

[79] Chamorro V., Morales-Cano D., Milara J., Barreira B., Moreno L., Callejo M., Mondejar-Parreño G., Esquivel-Ruiz S., Cortijo J., Cogolludo Á. (2018). Riociguat versus sildenafil on hypoxic pulmonary vasoconstriction and ventilation/perfusion matching. PLoS ONE.

[80] Johnson K.A. (2007). Imaging techniques for small animal imaging models of pulmonary disease: Micro-CT. Toxicol. Pathol..

[81] Yu W., Mi L., Long T. (2017). Efficacies of rosiglitazone and retinoin on bleomycin-induced pulmonary fibrosis in rats. Exp. Ther. Med..

[82] Yu W.C., Tian L.Y., Cheng W. (2015). Efficacy study of edaravone and acetylcysteine towards bleomycin-induced rat pulmonary fibrosis. Int. J. Clin. Exp. Med..

[83] Chagnon F., Fournier C., Charette P.G., Moleski L., Payet M.D., Dobbs L.G., Lesur O. (2010). In vivo intravital endoscopic confocal fluorescence microscopy of normal and acutely injured rat lungs. Lab. Investig..

[84] Egger C., Cannet C., Gérard C., Dunbar A., Tigani B., Beckmann N. (2015). Hyaluronidase modulates bleomycin-induced lung injury detected noninvasively in small rodents by radial proton MRI. J. Magn. Reson. Imaging.

[85] Egger C., Cannet C., Gérard C., Jarman E., Jarai G., Feige A., Suply T., Micard A., Dunbar A., Tigani B. (2013). Administration of Bleomycin via the Oropharyngeal Aspiration Route Leads to Sustained Lung Fibrosis in Mice and Rats as Quantified by UTE-MRI and Histology. PLoS ONE.

[86] Babin A.L., Cannet C., Gerard C., Saint-Mezard P., Page C.P., Sparrer H., Matsuguchi T., Beckmann N. (2012). Bleomycin-induced lung injury in mice investigated by MRI: Model assessment for target analysis. Magn. Reson. Med..

[87] Egger C., Cannet C.L., Gérard C., Suply T., Ksiazek I., Jarman E., Beckmann N. (2017). Effects of the fibroblast activation protein inhibitor, PT100, in a murine model of pulmonary fibrosis. Eur. J. Pharmacol..

[88] Hodono S., Shimokawa A., Stewart N.J., Yamauchi Y., Nishimori R., Yamane M., Ima H., Fujiwara H., Kimura A. (2018). Ethyl Pyruvate Improves Pulmonary Function in Mice with Bleomycin-induced Lung Injury as Monitored with Hyperpolarized 129Xe MR Imaging. Magn. Reson. Med. Sci..

[89] Shea B.S., Probst C.K., Brazee P.L., Rotile N.J., Blasi F., Weinreb P.H., Black K.E., Sosnovik D.E., Van Cott E.M., Violette S.M. (2017). Uncoupling of the profibrotic and hemostatic effects of thrombin in lung fibrosis. JCI Insight.

[90] Waghorn P.A., Jones M.C.M., Rotile N.J., Koerner S.K., Ferreira D.S., Chen H.H., Probst C.K., Tager A.M., Caravan P. (2017). Molecular Magnetic Resonance Imaging of Lung Fibrogenesis with an Oxyamine-Based Probe. Angew. Chem. Int. Ed. Engl..

[91] Tassali N., Bianchi A., Lux F., Raffard G., Sanchez S., Tillement O., Crémillieux Y. (2016). MR imaging, targeting and characterization of pulmonary fibrosis using intra-tracheal administration of gadolinium-based nanoparticles. Contrast Media Mol. Imaging.

[92] John A.E., Luckett J.C., Tatler A.L., Awais R.O., Desai A., Habgood A., Ludbrook S., Blanchard A.D., Perkins A.C., Jenkins R.G. (2013). Preclinical SPECT/CT imaging of alphavbeta6 integrins for molecular stratification of idiopathic pulmonary fibrosis. J. Nucl. Med..

[93] Schniering J., Benešová M., Brunner M., Haller S., Cohrs S., Frauenfelder T., Vrugt B., Feghali-Bostwick C.A., Schibli R., Distler O. (2019). Visualisation of interstitial lung disease by molecular imaging of integrin αvβ3 and somatostatin receptor 2. Ann. Rheum. Dis..

[94] Schniering J., Guo L., Brunner M., Schibli R., Ye S., Distler O., Béhé M., Maurer B. (2018). Evaluation of 99mTc-rhAnnexin V-128 SPECT/CT as a diagnostic tool for early stages of interstitial lung disease associated with systemic sclerosis. Arthritis Res. Ther..

[95] Kelderhouse L.E., Robins M.T., Rosenbalm K.E., Hoylman E.K., Mahalingam S., Low P.S. (2015). Prediction of Response to Therapy for Autoimmune/Inflammatory Diseases Using an Activated Macrophage-Targeted Radioimaging Agent. Mol. Pharm..

[96] Schniering J., Borgna F., Siwowska K., Benesova M., Cohrs S., Hasler R., van der Meulen N.P., Maurer B., Schibli R., Müller C. (2018). In Vivo Labeling of Plasma Proteins for Imaging of Enhanced Vascular Permeability in the Lungs. Mol. Pharm..

[97] Jailkhani N., Ingram J.R., Rashidian M., Rickelt S., Tian C., Mak H., Jiang Z., Ploegh H.L., Hynes R.O. (2019). Noninvasive imaging of tumor progression, metastasis, and fibrosis using a nanobody targeting the extracellular matrix. Proc. Natl. Acad. Sci. USA.

[98] Ruscitti F., Ravanetti F., Donofrio G., Ridwan Y., van Heijningen P., Essers J., Villetti G., Cacchioli A., Vos W., Stellari F.F. (2018). A Multimodal Imaging Approach Based on Micro-CT and Fluorescence Molecular Tomography for Longitudinal Assessment of Bleomycin-Induced Lung Fibrosis in Mice. J. Vis. Exp..

[99] Kelderhouse L.E., Mahalingam S., Low P.S. (2016). Predicting Response to Therapy for Autoimmune and Inflammatory Diseases Using a Folate Receptor-Targeted Near-Infrared Fluorescent Imaging Agent. Mol. Imaging Biol..

[100] Cai Y., Zhu L., Zhang F., Niu G., Lee S., Kimura S., Chen X. (2013). Noninvasive monitoring of pulmonary fibrosis by targeting matrix metalloproteinases (MMPs). Mol. Pharm..

[101] Stellari F.F., Ruscitti F., Pompilio D., Ravanetti F., Tebaldi G., Macchi F., Verna A.E., Villetti G., Donofrio G. (2017). Heterologous matrix metalloproteinase gene promoter activity allows in vivo real-time imaging of bleomycin-induced lung fibrosis in transiently transgenized mice. Front. Immunol..

[102] Li W., Yang L., Fan W., Chen Y., Tian J., Ma L., Liu B., Li Y., Wang S., Fu Q. (2015). Luciferase expression is driven by the promoter of fibroblast activation protein-α in murine pulmonary fibrosis. Biotechnol. Lett..

[103] Buonfiglio L.G., Bagegni M., Borcherding J.A., Sieren J.C., Caraballo J.C., Reger A., Andrew Reger A., Zabner J., Li X., Comellas A.P. (2016). Protein Kinase Czeta Inhibitor Promotes Resolution of Bleomycin-Induced Acute Lung Injury. Am. J. Respir. Cell Mol. Biol..

[104] Cavanaugh D., Travis E.L., Price R.E., Gladish G., White R.A., Wang M., Cody D.D. (2006). Quantification of bleomycin-induced murine lung damage in vivo with micro-computed tomography. Acad. Radiol..

[105] Choi E.J., Jin G.Y., Bok S.M., Han Y.M., Lee Y.S., Jung M.J., Kwon K.S. (2014). Serial micro-CT assessment of the therapeutic effects of rosiglitazone in a bleomycin-induced lung fibrosis mouse model. Korean J. Radiol..

[106] Kimura T., Nojiri T., Hosoda H., Shintani Y., Inoue M., Miyazato M., Okumura M., Kangawa K. (2015). Exacerbation of bleomycin-induced injury by lipopolysaccharide in mice: Establishment of a mouse model for acute exacerbation of interstitial lung diseases. Eur. J. Cardiothorac. Surg..

[107] Shofer S., Badea C., Auerbach S., Schwartz D.A., Johnson G.A. (2007). A micro-computed tomography-based method for the measurement of pulmonary compliance in healthy and bleomycin-exposed mice. Exp. Lung Res..

[108] Vande Velde G., Poelmans J., De Langhe E., Hillen A., Vanoirbeek J., Himmelreich U., Loris R.J. (2016). Longitudinal micro-CT provides biomarkers of lung disease that can be used to assess the effect of therapy in preclinical mouse models, and reveal compensatory changes in lung volume. Dis. Model. Mech..

[109] Fu Q., Zheng Y., Dong X., Wang L., Jiang C.G. (2017). Activation of cannabinoid receptor type 2 by JWH133 alleviates bleomycin-induced pulmonary fibrosis in mice. Oncotarget.

[110] Shao R., Wang F.J., Lyu M., Yang J., Zhang P., Zhu Y. (2019). Ability to Suppress TGF-beta-Activated Myofibroblast Differentiation Distinguishes the Anti-pulmonary Fibrosis Efficacy of Two Danshen-Containing Chinese Herbal Medicine Prescriptions. Front. Pharmacol..

[111] Arora A., Bhuria V., Hazari P.P., Pathak U., Mathur S., Roy B.G., Soni R., Dwarakanath B.S., Bhatt A.N. (2018). Amifostine analog, DRDE-30, attenuates bleomycin-induced pulmonary fibrosis in mice. Front. Pharmacol..

[112] Povedano J.M., Martinez P., Serrano R., Tejera A., Gómez-López G., Bobadilla M., Flores J.M., Bosch F., Blasco M.A. (2018). Therapeutic effects of telomerase in mice with pulmonary fibrosis induced by damage to the lungs and short telomeres. Elife.

[113] Hellbach K., Yaroshenko A.L., Willer K., Conlon T.M., Braunagel M.B., Auweter S., Yildirim A.Ö., Eickelberg O., Pfeiffer F., Reiser M.F. (2017). X-ray dark-field radiography facilitates the diagnosis of pulmonary fibrosis in a mouse model. Sci. Rep..

[114] Wynn T.A. (2011). Integrating mechanisms of pulmonary fibrosis. J. Exp. Med..

[115] Cox T.R., Erler J.T. (2011). Remodeling and homeostasis of the extracellular matrix: Implications for fibrotic diseases and cancer. Dis. Model. Mech..

[116] Thannickal V.J., Toews G.B., White E.S., Lynch J.P., Martinez F.J. (2004). Mechanisms of pulmonary fibrosis. Annu. Rev. Med..

[117] Das D.K., Steinberg H., Bandyopadhyay D., Hoory S. (1988). Potential use of indium-111-labeled polymorphonuclear leukocytes for the detection of lung microvascular injury. J. Nucl. Med..

[118] Hill L.L., Chen D.L., Kozlowski J., Schuster D.P. (2004). Neutrophils and neutrophil products do not mediate pulmonary hemodynamic effects of endotoxin on oleic acid-induced lung injury. Anesth. Analg..

[119] Richard J.C., Pouzot C., Gros A., Tourevieille C., Lebars D., Lavenne F., Frerichs I., Guerin C. (2009). Electrical impedance tomography compared to positron emission tomography for the measurement of regional lung ventilation: An experimental study. Crit. Care.

[120] Medhora M., Haworth S., Liu Y., Narayanan J., Gao F., Zhao M., Audi S., Jacobs E.R., Fish B.L., Clough A.V. (2016). Biomarkers for Radiation Pneumonitis Using Noninvasive Molecular Imaging. J. Nucl. Med..

[121] Driscoll K.E., Costa D.L., Hatch G., Henderson R., Oberdorster G., Salem H., Schlesinger R.B. (2000). Intratracheal instillation as an exposure technique for the evaluation of respiratory tract toxicity: Uses and limitations. Toxicol. Sci..

[122] Vande Velde G., De Langhe E., Poelmans J., Bruyndonckx P., d’Agostino E., Verbeken E., Bogaerts R., Lories R., Himmelreich U. (2015). Longitudinal in vivo microcomputed tomography of mouse lungs: No evidence for radiotoxicity. Am. J. Physiol. Lung Cell. Mol. Physiol..

[123] Barbayianni I., Ninou I., Tzouvelekis A., Aidinis V. (2018). Bleomycin Revisited: A Direct Comparison of the Intratracheal Micro-Spraying and the Oropharyngeal Aspiration Routes of Bleomycin Administration in Mice. Front. Med. (Lausanne).

[124] Luzina I.G., Todd N.W., Sundararajan S., Atamas S.P. (2015). The cytokines of pulmonary fibrosis: Much learned, much more to learn. Cytokine.

[125] Gauldie J., Kolb M. (2008). Animal models of pulmonary fibrosis: How far from effective reality?. Am. J. Physiol. Lung Cell. Mol. Physiol..

[126] Mahmutovic Persson I., Falk Hakansson H., Orbom A., Liu J., von Wachenfeldt K., Olsson L.E. (2020). Imaging Biomarkers and Pathobiological Profiling in a Rat Model of Drug-Induced Interstitial Lung Disease Induced by Bleomycin. Front. Physiol..

[127] Koo L.C., Clark J.A., Quesenberry C.P., Higenbottam T., Nyberg F., Wolf M.K., Steinberg M.H., Forsythe B.H. (2005). National differences in reporting ‘pneumonia’ and ‘pneumonia interstitial’: An analysis of the WHO International Drug Monitoring Database on 15 drugs in nine countries for seven pulmonary conditions. Pharm. Drug Saf..

