# Supplementary material for: Imaging Biomarkers in Animal Models of Drug-Induced Lung Injury: A Systematic Review
Source: J Clin Med. 2020 Dec 30;10(1):107. doi: 10.3390/jcm10010107 (PMC7795017; doi:10.3390/jcm10010107)
Supplement: Supplementary file 1 [file jcm-10-00107-s001.zip › Supplementary files/Additional file 1.docx]

**Additional file 1: The four search categories with corresponding search terms.**

***Animal models***

animal model'/exp OR 'animal model' OR 'animal models' OR 'disease model'/exp OR 'disease model' OR 'disease models' OR rat OR rats OR rodent OR rodents OR mouse OR mice OR murine OR rabbit* OR 'guinea pig‘ OR 'guinea pigs' OR hamster OR hamsters OR 'rat'/exp OR 'mouse'/exp OR 'rodent'/exp OR 'murine'/exp OR 'rabbit'/exp OR 'guinea pig'/exp OR 'hamster'/exp

***Imaging***

Imaging OR imaging/exp OR ct OR hrct OR 'computed tomography' OR ‘computer assisted tomography’/exp OR radiography OR radiography/exp OR 'magnetic resonance' OR mri OR ‘nuclear magnetic resonance imaging’/exp OR 'pet-ct' OR 'pet-mri scanner' OR 'positron emission tomography' OR pet OR ‘positron emission tomography’/exp OR 'near infrared' OR ‘near infrared spectroscopy’/exp OR ultrasound OR ultrasound/exp OR ultrasonography OR 'single photon emission computed tomography'/exp OR 'single photon emission computed tomography' OR 'spect' OR 'optical imaging'/exp OR 'optical imaging' OR 'fluorescence' OR 'luminescence' OR 'bioluminescence'

***Disease***

'hypersensitive pneumonitis' OR 'pneumonia'/exp OR pneumonia OR 'organizing pneumonia' OR 'diffuse alveolar damage' OR 'nonspecific interstitial pneumonia' OR nsip OR 'eosinophilic pneumonia' OR 'bronchiolitis obliterans' OR boop OR 'pulmonary haemorrhage'/exp OR 'pulmonary haemorrhage' OR 'granulomatous pneumonitis' OR 'emphysema'/exp OR emphysema OR 'airflow obstruction'/exp OR 'airflow obstruction' OR 'alveolar hypoventilation'/exp OR 'alveolar hypoventilation' OR 'respiratory muscle dysfunction' OR 'non-cardiogenic pulmonary oedema'/exp OR 'non-cardiogenic pulmonary oedema' OR 'noncardiogenic lung edema'/exp OR 'noncardiogenic lung edema' OR 'alveolar haemorrhage'/exp OR 'alveolar haemorrhage' OR 'lung hemorrhage'/exp OR 'lung hemorrhage' OR 'diffuse lung processes' OR 'acute respiratory distress syndrome'/exp OR ard OR 'acute respiratory distress syndrome' OR 'metastatic calcification'/exp OR 'metastatic calcification' OR 'nodular infiltrates' OR 'pulmonary hypertension'/exp OR 'pulmonary hypertension' OR 'pulmonary artery hypertension' OR 'veno-occlusive disease'/exp OR 'veno-occlusive disease' OR 'thrombotic microangiopathy'/exp OR 'thrombotic microangiopathy' OR 'thrombotic micro angiopathy' OR 'interstitial pneumonia-like pattern' OR 'cellular interstitial infiltrates' OR 'interstitial pneumonia'/exp OR 'interstitial pneumonia' OR 'small vessel angiitis' OR 'pulmonary veno-occlusive disease'/exp OR 'pulmonary veno-occlusive disease' OR 'thromboembolism'/exp OR thromboembolism OR 'vasculitis'/exp OR vasculitis OR 'alveolar processes' OR 'eosinophilic syndromes' OR 'systemic lupus erythematosus–like' OR 'sle-like' OR 'drug induced lung injury' OR 'drug induced interstitial lung disease' OR diild OR 'interstitial lung diseases' OR 'interstitial lung disease‘ OR 'lung injury' OR 'fibrosis'

***Drugs***

'Antimicrobial Agents' OR 'Amphotericin B' OR 'Isoniazid' OR 'Nitrofurantoin' OR 'Sulfasalazine' OR 'Anti-Inflammatory Agents' OR 'Aspirin' OR 'Etanercept' OR 'Gold' OR 'Infliximab' OR 'Methotrexate' OR 'Nonsteroidal anti-inflammatory drugs' OR 'Penicillamine' OR 'Biological Agents' OR 'Adalimumab' OR 'Alemtuzumab' OR 'Bevacizumab' OR 'Cetuximab' OR 'Rituximab' OR 'Trastuzumab' OR 'Tumor necrosis factor (TNF)-α blockers' OR 'Cardiovascular Agents' OR 'ACE inhibitors' OR 'Amiodarone' OR 'Anticoagulants' OR 'ß-Blockers' OR 'Flecainide' OR 'Hydrochlorothiazide' OR 'Procainamide' OR 'Statins' OR 'Tocainide' OR 'Chemotherapeutic Agents' OR 'Azathioprine' OR 'BCNU' OR 'Chemotherapeutic Agents' OR 'Bleomycin' OR 'Bortezomib' OR 'Busulfan' OR 'Carmustine' OR 'Chlorambucil' OR 'Colony-stimulating factors' OR 'Cyclophosphamide' OR 'Cytarabine' OR 'Deferoxamine' OR 'Docetaxel' OR 'Doxorubicin' OR 'Erlotinib' OR 'Etoposide' OR 'Fludarabine' OR 'Flutamide' OR 'Gefitinib' OR 'Gemcitabine' OR 'Hydroxyurea' OR 'Imatinib' OR 'Interferons' OR 'Lomustine' OR 'Melphalan' OR 'Methotrexate' OR 'Methyl-CCNU' OR 'Mitomycin-C' OR 'Nitrosoureas' OR 'Paclitaxel' OR 'Procarbazine' OR 'Thalidomide' OR 'Vinblastine' OR 'Zinostatin' OR 'Miscellanous' OR 'Bromocriptine' OR 'Carbamazepine' OR 'Cabergolide' OR 'Methysergide' OR 'Penicillamine' OR 'Phenytoin' OR 'Sirolimus' OR 'Talc'
