# Supplementary material for: Imaging Biomarkers in Animal Models of Drug-Induced Lung Injury: A Systematic Review
Source: J Clin Med. 2020 Dec 30;10(1):107. doi: 10.3390/jcm10010107 (PMC7795017; doi:10.3390/jcm10010107)
Supplement: Supplementary file 1 [file jcm-10-00107-s001.zip › Supplementary files/Additional file 2.docx]

**Additional file 2:** Remaining description of the Material and Methods section with two tables. Table 1 summarises the “Headings for data extraction”, and Table 2 demonstrates the eligibility criteria for excluded articles.

***The survey process, risk of bias and quality score of selected papers***

After the eligibility process, the remaining articles underwent selection process and data extraction. The final number of articles selected for data extraction were 182 and can be found in alphabetic order in Additional file 3. These 182 articles were also scored by the investigating authors. The score was based on 3 different questions on whether the surveyed article possessed any translational value and if this study approach or layout is of importance for continuing with potential drug-induced injury examination in patients. Secondly, the imaging technique and the procedure was evaluated in a translational setting and whether it could be applied in a potential clinical setting. Lastly the DIILD model as such was judged on how it was executed and the value of the study outcome in relation to the study aim. Each article was given 0-3 scoring points on each of the three questions, resulting in a score from 0 to maximum 9. In this way the authors were able to set more focus on the most translationally valuable papers for further discussion and future engaging of potential study design. The risk of scoring bias was discussed and the differences in opinion were resolved by consensus method.

***Study selection* *and data collection process***

Extracted data from each of the 182 articles was collected and summarised in an integrated table, from which the data was analysed and presented. The type of data that was extracted from each article was stated by headings that all authors agreed on beforehand, presented in Table 1 here in Additional file 2. All reviewers extracted at least 20 articles each and consensus was reached through several meetings and discussions for optimal data extraction strategy. After a first round of reviewing, the authors made adjustments to the headings and defined in more detail what should be the content of the variables included in the extracted data from each article. Those articles that passes the eligibility criteria (in total 227+57) but were not selected in the final step for data extraction (in total 85+15) are listed in Table 2, here in Additional file 2, based on the exclusion criteria.

**Table 1;** Additional file 2: Headings for data extraction of all selected articles.

| **Headings for data extraction:** |  |
| --- | --- |
|  | Reference information |
|  | Reviewer |
|  | Should this paper be excluded from the data base (Y/N) |
|  | Does this paper explicitly express DIILD relevance? (Y/P/N) |
| **Animal model** | Species |
|  | Strain |
|  | Sex |
|  | Age |
|  | Weight |
|  | #of animals |
|  | Model |
|  | Inducing agent |
|  | Pathophysiology |
|  | Administration route |
|  | Dose |
|  | Intervention |
| **Imaging** | Modality |
|  | Imaging (type, sequence, scan) |
|  | Contrast agent |
|  | Administration |
|  | Imaging concept |
|  | Tracer |
|  | Multi-imaging (multi modalities) |
|  | Duration of the model and imaging |
|  | Number of imaging events |
| **Analysis** | Readout (the signal, CT HU increase etc) |
|  | Quantification |
|  | Endpoints (Volume measure in MRI..) |
| **Animal monitoring** | Anaesthesia |
|  | Respiration |
|  | Triggered imaging |
| **Validation** | Histology |
|  | Biomarker (non-imaging) |
| **Translational relevance** | Could this imaging setting be transferred to human setting |
|  | Imaging-pathology correlation? |
|  | Correlation at same time points between imaging, lung function or any other biomarker? |
|  | Longitudinal studies |
|  | Reversibility or treatment effects |
|  | Correlation with outcomes (outcome that can have a predictive value?) |
| **Scoring** | Translational relevance (animal model) |
|  | Translational possibility (imaging modality) |
|  | DIILD relevant |
|  | Total Score |

| **Initial search** |  |  |
| --- | --- | --- |
| **Eligibility criteria applied for article selection** | **Number of articles** | **Percentage (%)** |
| Not live imaging | 30 | 35.3 |
| Not DIILD or lung injury model | 19 | 22.4 |
| Not containing imaging | 13 | 15.3 |
| Review article, not original data | 13 | 15.3 |
| Not lung focused imaging, other organs imaged | 9 | 10.6 |
| Human studies | 1 | 1.2 |
| Total | 85 | 100 |

**Table 2;** Additional file 2: Eligibility criteria applied for exclusion of the 85 articles excluded in the review.

| **Follow-up search** |  |  |
| --- | --- | --- |
| **Eligibility criteria applied for article selection** | **Number of articles** | **Percentage (%)** |
| Not live imaging | 8 | 47.0 |
| Not containing imaging | 1 | 5.9 |
| Review article, not original data | 1 | 5.9 |
| Not lung focused imaging, other organs imaged | 6 | 35.3 |
| Invasive imaging | 1 | 5.9 |
| Total | 17 | 100 |
