# Supplementary material for: Imaging Biomarkers in Animal Models of Drug-Induced Lung Injury: A Systematic Review
Source: J Clin Med. 2020 Dec 30;10(1):107. doi: 10.3390/jcm10010107 (PMC7795017; doi:10.3390/jcm10010107)
Supplement: Supplementary file 1 [file jcm-10-00107-s001.zip › Supplementary files/Additional file 3.docx]

**Additional file 3:** The 182 selected articles from which data was extracted and presented in this review. The references are listed in alphabetic order.

**Ackermann M**, Kim YO, Wagner WL, Schuppan D, Valenzuela CD, Mentzer SJ, et al. (**2017**). Effects of nintedanib on the microvascular architecture in a lung fibrosis model. Angiogenesis: 1-14.

**Arora A**, Bhuria V, Hazari P. P, Pathak U, Mathur S, Roy B. G, et al. (**2018**). Amifostine analog, DRDE-30, attenuates bleomycin-induced pulmonary fibrosis in mice. Front Pharmacol. 9:394.

**Audi SH**, Jacobs ER, Zhao M, Roerig DL, Haworth ST, & Clough AV (**2015**). In vivo detection of hyperoxia-induced pulmonary endothelial cell death using (99m)Tc-duramycin. Nuclear medicine and biology 42: 46-52.

**Babin AL**, Cannet C, Gerard C, Saint-Mezard P, Page CP, Sparrer H, et al. (**2012**). Bleomycin-induced lung injury in mice investigated by MRI: model assessment for target analysis. Magn Reson Med 67: 499-509.

**Babin AL**, Cannet C, Gérard C, Wyss D, Page CP, & Beckmann N (**2011**). Noninvasive assessment of bleomycin-induced lung injury and the effects of short-term glucocorticosteroid treatment in rats using MRI. Journal of Magnetic Resonance Imaging 33: 603-614.

**Bai KJ**, Chuang KJ, Chen JK, Hua HE, Shen YL, Liao WN, et al. (**2017**). Investigation into the pulmonary inflammopathology of exposure to nickel oxide nanoparticles in mice. Nanomedicine.

**Balazs G**, Noma S, Khan A, Eacobacci T, & Herman PG (**1994**). Bleomycin-induced fibrosis in pigs: evaluation with CT. Radiology 191: 269-272.

**Barabasch A**, von Stillfried S, Kuhl C. K, Heinzel A, Sander A, Isfort P. (**2019**). Toward Transpulmonary Chemoembolization with Degradable Starch Microspheres: Systematic Analysis of Local and Systemic Effects in a Porcine Model. CardioVascular and Interventional Radiology, 42(10):1475-1482.

**Bianchi A**, Tibiletti M, Kjorstad A, Birk G, Schad LR, Stierstorfer B, et al. (**2015**a). Three-dimensional accurate detection of lung emphysema in rats using ultra-short and zero echo time MRI. NMR Biomed 28: 1471-1479.

**Bianchi A**, Tibiletti M, Kjorstad A, Birk G, Schad LR, Stierstorfer B, et al. (**2015**b). Functional Proton MRI in Emphysematous Rats. Invest Radiol 50: 812-820.

**Birukova AA**, Meng F, Tian Y, Meliton A, Sarich N, Quilliam LA, et al. (**2015**). Prostacyclin post-treatment improves LPS-induced acute lung injury and endothelial barrier recovery via Rap1. Biochim Biophys Acta 1852: 778-791.

**Blank DA**, Hooper SB, Binder-Heschl C, Kluckow M, Gill AW, LaRosa DA, et al. (**2016**). Lung ultrasound accurately detects pneumothorax in a preterm newborn lamb model. J Paediatr Child Health 52: 643-648.

**Bondue B**, Castiaux A, Van Simaeys G, Mathey C, Sherer F, Egrise D, et al. (**2019**). Absence of early metabolic response assessed by 18F-FDG PET/CT after initiation of antifibrotic drugs in IPF patients. Respiratory Research, 15;20(1):10.

**Bondue B**, Sherer F, Van Simaeys G, Doumont G, Egrise D, Yakoub Y, et al. (**2015**). PET/CT with 18F-FDG- and 18F-FBEM-labeled leukocytes for metabolic activity and leukocyte recruitment monitoring in a mouse model of pulmonary fibrosis. Journal of Nuclear Medicine 56: 127-132.

**Borgmann S**, Schmidt J, Goebel U, Haberstroh J, Guttmann J, Schumann S, et al. (**2018**). Dorsal recruitment with flow-controlled expiration (FLEX): An experimental study in mechanically ventilated lung-healthy and lung-injured pigs. Critical Care 22(1):245.

**Bunskoek PE**, Seyedmousavi S, Gans SJ, van Vierzen PB, Melchers WJ, van Elk CE, et al. (**2017**). Successful treatment of azole-resistant invasive aspergillosis in a bottlenose dolphin with high-dose posaconazole. Med Mycol Case Rep 16: 16-19.

**Buonfiglio LG**, Bagegni M, Borcherding JA, Sieren JC, Caraballo JC, Reger A, et al. (**2016**). Protein Kinase Czeta Inhibitor Promotes Resolution of Bleomycin-Induced Acute Lung Injury. Am J Respir Cell Mol Biol 55: 869-877.

**Cai Y**, Zhu L, Zhang F, Niu G, Lee S, Kimura S, et al. (**2013**). Noninvasive monitoring of pulmonary fibrosis by targeting matrix metalloproteinases (MMPs). Mol Pharm 10: 2237-2247.

**Capa Kaya G**, Bekis R, Kirimca F, Ertay T, Kargi A, Gure A, et al. (**2001**). Use of technetium-99m HMPAO scintigraphy for the detection of amiodarone lung toxicity in a rabbit model. Eur J Nucl Med 28: 346-350.

**Caravan P**, Yang Y, Zachariah R, Schmitt A, Mino-Kenudson M, Chen HH, et al. (**2013**). Molecular magnetic resonance imaging of pulmonary fibrosis in mice. Am J Respir Cell Mol Biol 49: 1120-1126.

**Cavanaugh D**, Travis EL, Price RE, Gladish G, White RA, Wang M, et al. (**2006**). Quantification of bleomycin-induced murine lung damage in vivo with micro-computed tomography. Acad Radiol 13: 1505-1512.

**Ceelen JJM**, Schols A, van Hoof SJ, de Theije CC, Verhaegen F, & Langen RCJ (**2017**). Differential regulation of muscle protein turnover in response to emphysema and acute pulmonary inflammation. Respir Res 18: 75.

**Chagnon F**, Fournier C, Charette PG, Moleski L, Payet MD, Dobbs LG, et al. (**2010**). In vivo intravital endoscopic confocal fluorescence microscopy of normal and acutely injured rat lungs. Lab Invest 90: 824-834.

**Chamorro V**, Morales-Cano D, Milara J, Barreira B, Moreno L, Callejo M, et al. (**2019**). Riociguat versus sildenafil on hypoxic pulmonary vasoconstriction and ventilation/perfusion matching. PLoS One; Comparative Study.

**Chen DL**, Mintun MA, & Schuster DP (**2004**). Comparison of methods to quantitate 18F-FDG uptake with PET during experimental acute lung injury. J Nucl Med 45: 1583-1590.

**Cho WS**, Cho M, Kim SR, Choi M, Lee JY, Han BS, et al. (**2009**). Pulmonary toxicity and kinetic study of Cy5.5-conjugated superparamagnetic iron oxide nanoparticles by optical imaging. Toxicol Appl Pharmacol 239: 106-115.

**Choi EJ**, Jin GY, Bok SM, Han YM, Lee YS, Jung MJ, et al. (**2014**). Serial micro-CT assessment of the therapeutic effects of rosiglitazone in a bleomycin-induced lung fibrosis mouse model. Korean J Radiol 15: 448-455.

**Cleveland ZI**, Virgincar RS, Qi Y, Robertson SH, Degan S, & Driehuys B (**2014**). 3D MRI of impaired hyperpolarized 129Xe uptake in a rat model of pulmonary fibrosis. NMR Biomed 27: 1502-1514.

**Cleveland ZI**, Zhou YM, Akinyi TG, Dunn RS, Davidson CR, Guo J, et al. (**2017**). Magnetic resonance imaging of disease progression and resolution in a transgenic mouse model of pulmonary fibrosis. Am J Physiol Lung Cell Mol Physiol 312: L488-L499.

**Cline J.M**, Dugan G, Bourland J.D, Perry D.L, Stitzel J.D, Weaver A.A, et al. (**2018**). Post-Irradiation Treatment with a Superoxide Dismutase Mimic, MnTnHex-2-PyP(5+), Mitigates Radiation Injury in the Lungs of Non-Human Primates after Whole-Thorax Exposure to Ionizing Radiation. Antioxidants (Basel) 7(3):40.

**Clough AV**, Audi SH, Haworth ST, & Roerig DL (**2012**). Differential lung uptake of 99mTc-hexamethylpropyleneamine oxime and 99mTc-duramycin in the chronic hyperoxia rat model. J Nucl Med 53: 1984-1991.

**Couch MJ**, Fox MS, Viel C, Gajawada G, Li T, Ouriadov AV, et al. (**2016**). Fractional ventilation mapping using inert fluorinated gas MRI in rat models of inflammation and fibrosis. NMR Biomed 29: 545-552.

**Cui P**, Feng X, Zhu F, Yao Y, Xiao S, Gong Z, et al. (**2018**). Acute respiratory distress syndrome induced by white smoke inhalation: A potential animal model for evaluating pathological changes and underlying mechanisms. Cell Physiol Biochem. 47(6):2396-2406.

**Cui P**, Xin H, Yao Y, Xiao S, Zhu F, Gong Z, et al. (**2018**). Human amnion-derived mesenchymal stem cells alleviate lung injury induced by white smoke inhalation in rats. Stem Cell Res Ther. 9(1):101.

**Dadrich M**, Nicolay NH, Flechsig P, Bickelhaupt S, Hoeltgen L, Roeder F, et al. (**2016**). Combined inhibition of TGFbeta and PDGF signaling attenuates radiation-induced pulmonary fibrosis. Oncoimmunology 5: e1123366.

**Das DK**, Bandyopadhyay D, Hoory S, & Steinberg H (**1988**). Role of polymorphonuclear leukocytes in hyperoxic lung injury. Prevention of neutrophil influx into the lung endothelium during oxygen exposure by ibuprofen. Biomed Biochim Acta 47: 1023-1036.

**Das DK**, Steinberg H, Bandyopadhyay D, & Hoory S (**1988**). Potential use of indium-111-labeled polymorphonuclear leukocytes for the detection of lung microvascular injury. J Nucl Med 29: 657-662.

**De Langhe E**, Vande Velde G, Hostens J, Himmelreich U, Nemery B, Luyten FP, et al. (**2012**). Quantification of lung fibrosis and emphysema in mice using automated micro-computed tomography. PLoS One 7: e43123.

**De Oliveira M. V**, De Novaes Rocha N, Santos R. S, Macedo Rocco M. R, De Magalhães R. F, Silva J. D, et al. (**2019**). Endotoxin-induced emphysema exacerbation: A novel model of chronic obstructive pulmonary disease exacerbations causing cardiopulmonary impairment and diaphragm dysfunction. Front Physiol 10:664.

**De Ruysscher D**, Granton PV, Lieuwes NG, van Hoof S, Wollin L, Weynand B, et al. (**2017**). Nintedanib reduces radiation-induced microscopic lung fibrosis but this cannot be monitored by CT imaging: A preclinical study with a high precision image-guided irradiator. Radiother Oncol 124: 482-487.

**Desogere P**, Tapias LF, Hariri LP, Rotile NJ, Rietz TA, Probst CK, et al. (**2017**a). Type I collagen-targeted PET probe for pulmonary fibrosis detection and staging in preclinical models. Sci Transl Med 9.

**Desogere P**, Tapias LF, Rietz TA, Rotile N, Blasi F, Day H, et al. (**2017**b). Optimization of a Collagen-Targeted PET Probe for Molecular Imaging of Pulmonary Fibrosis. J Nucl Med 58: 1991-1996.

**Dhanani J. A**, Cohen J, Parker S. L, Chan H. K, Tang P, Ahern B. J, et al. (**2018**). A research pathway for the study of the delivery and disposition of nebulised antibiotics: an incremental approach from in vitro to large animal models. Intensive Care Med Exp 6(1):17.

**Drozd K**, Ahmadi A, Deng Y, Jiang B, Petryk J, Thorn S, et al. (**2017**). Effects of an endothelin receptor antagonist, Macitentan, on right ventricular substrate utilization and function in a Sugen 5416/hypoxia rat model of severe pulmonary arterial hypertension. J Nucl Cardiol 24: 1979-1989.

**Durmus-Altun G**, Altun A, Sami Salihoglu Y, Altaner S, Berkada S, & Ozbay G (**2004**). Value of technetium-99m diethyltriamine pentaaceticacid radioaerosol inhalation lung scintigraphy for the stage of amiodarone-induced pulmonary toxicity. Int J Cardiol 95: 193-197.

**Egger C**, Cannet C, Gérard C, Dunbar A, Tigani B, & Beckmann N (**2015**). Hyaluronidase modulates bleomycin-induced lung injury detected noninvasively in small rodents by radial proton MRI. Journal of Magnetic Resonance Imaging 41: 755-764.

**Egger C**, Cannet C, Gérard C, Jarman E, Jarai G, Feige A, et al. (**2013**). Administration of Bleomycin via the Oropharyngeal Aspiration Route Leads to Sustained Lung Fibrosis in Mice and Rats as Quantified by UTE-MRI and Histology. PLoS ONE 8.

**Egger C**, Cannet C, Gérard C, Suply T, Ksiazek I, Jarman E, et al. (**2017**). Effects of the fibroblast activation protein inhibitor, PT100, in a murine model of pulmonary fibrosis. European Journal of Pharmacology 809: 64-72.

**Egger C**, Gerard C, Vidotto N, Accart N, Cannet C, Dunbar A, et al. (**2014**). Lung volume quantified by MRI reflects extracellular-matrix deposition and altered pulmonary function in bleomycin models of fibrosis: effects of SOM230. Am J Physiol Lung Cell Mol Physiol 306: L1064-1077.

**Fishman JA**, Strauss HW, Fischman AJ, Nedelman M, Callahan R, Khaw BA, et al. (**1991**). Imaging of Pneumocystis carinii pneumonia with 111In-labelled non-specific polyclonal IgG: an experimental study in rats. Nucl Med Commun 12: 175-187.

**Frerichs I**, Dargaville P. A, Rimensberger P. C. (**2019**). Regional pulmonary effects of bronchoalveolar lavage procedure determined by electrical impedance tomography. Intensive Care Med Exp. 7(1):11.

**Fu Q**, Zheng Y, Dong X, Wang L, & Jiang CG (**2017**). Activation of cannabinoid receptor type 2 by JWH133 alleviates bleomycin-induced pulmonary fibrosis in mice. Oncotarget 8: 103486-103498.

**Gao C**, Fujinawa R, Yoshida T, Ueno M, Ota F, Kizuka Y, et al. (**2017**). A keratan sulfate disaccharide prevents inflammation and the progression of emphysema in murine models. Am J Physiol Lung Cell Mol Physiol 312: L268-L276.

**Garofalo M**, Bennett A, Farese AM, Harper J, Ward A, Taylor-Howell C, et al. (**2014**a). The delayed pulmonary syndrome following acute high-dose irradiation: a rhesus macaque model. Health Phys 106: 56-72.

**Garofalo MC**, Ward AA, Farese AM, Bennett A, Taylor-Howell C, Cui W, et al. (**2014**b). A pilot study in rhesus macaques to assess the treatment efficacy of a small molecular weight catalytic metalloporphyrin antioxidant (AEOL 10150) in mitigating radiation-induced lung damage. Health Phys 106: 73-83.

**Ghobadi G**, Hogeweg LE, Faber H, Tukker WG, Schippers JM, Brandenburg S, et al. (**2010**). Quantifying local radiation-induced lung damage from computed tomography. Int J Radiat Oncol Biol Phys 76: 548-556.

**Ghoneim HE**, & McCullers JA (**2014**). Adjunctive corticosteroid therapy improves lung immunopathology and survival during severe secondary pneumococcal pneumonia in mice. J Infect Dis 209: 1459-1468.

**Goldklang MP**, Tekabe Y, Zelonina T, Trischler J, Xiao R, Stearns K, et al. (**2016**). Single-Photon Emission Computed Tomography/Computed Tomography Imaging in a Rabbit Model of Emphysema Reveals Ongoing Apoptosis In Vivo. Am J Respir Cell Mol Biol 55: 848-857.

**Grasso S**, Stripoli T, Mazzone P, Pezzuto M, Lacitignola L, Centonze P, et al. (**2014**). Low respiratory rate plus minimally invasive extracorporeal Co2 removal decreases systemic and pulmonary inflammatory mediators in experimental Acute Respiratory Distress Syndrome. Crit Care Med 42: e451-460.

**Guenthart B.A**, O’Neill J.D, Kim J, Queen D, Chicotka S, Fung, K, et al. (**2019**). Regeneration of severely damaged lungs using an interventional cross-circulation platform. Nat Commun. 10(1):1985.

**Gunther A**, Lubke N, Ermert M, Schermuly RT, Weissmann N, Breithecker A, et al. (**2003**). Prevention of bleomycin-induced lung fibrosis by aerosolization of heparin or urokinase in rabbits. Am J Respir Crit Care Med 168: 1358-1365.

**Guo J**, Hardie W.D, Cleveland Z.I, Davidson C, Xu X, Madala S.K, et al. (**2019**). Longitudinal free-breathing MRI measurement of murine lung physiology in a progressive model of lung fibrosis. J Appl Physiol. 126(4):1138-1149.

**Guo Y**, Ying S, Zhao X, Liu J, Wang Y. (**2019**). Increased expression of lung TRPV1/TRPA1 in a cough model of bleomycin-induced pulmonary fibrosis in Guinea pigs. BMC Pulm Med. 19(1):27.

**Gur D**, Glinert I, Aftalion M, Vagima Y, Levy Y, Rotem S, et al. (**2018**). Inhalational gentamicin treatment is effective against Pneumonic Plague In A Mouse Model. Front Microbiol. 9:741.

**Hadina S**, Wohlford-Lenane CL, & Thorne PS (**2012**). Comparison of in vivo bioluminescence imaging and lavage biomarkers to assess pulmonary inflammation. Toxicology 291: 133-138.

**Hagawane TN**, Gaikwad RV, & Kshirsagar NA (**2016**). Dual hit lipopolysaccharide & oleic acid combination induced rat model of acute lung injury/acute respiratory distress syndrome. Indian J Med Res 143: 624-632.

**Hagawane TN**, Mahuvakar AM, Gaikwad RV, & Kshirsagar NA (**2014**). Intratracheal (IT) Lipopolysaccharide (LPS) Induced acute lung injury (ALI)/Acute respiratory distress syndrome (ARDS) in wistar rats. Research Journal of Pharmacy and Technology 7: 419-424.

**Haslett C**, Shen AS, Feldsien DC, Allen D, Henson PM, & Cherniack RM (**1989**). 111Indium-labeled neutrophil migration into the lungs of bleomycin-treated rabbits assessed noninvasively by external scintigraphy. Am Rev Respir Dis 140: 756-763.

**Hatori A**, Yui J, Yamasaki T, Xie L, Kumata K, Fujinaga M, et al. (**2012**). PET imaging of lung inflammation with [18F]FEDAC, a radioligand for translocator protein (18 kDa). PLoS One 7: e45065.

**Hellbach K**, Yaroshenko A, Willer K, Conlon TM, Braunagel MB, Auweter S, et al. (**2017**). X-ray dark-field radiography facilitates the diagnosis of pulmonary fibrosis in a mouse model. Sci Rep 7: 340.

**Heuer JF**, Pelosi P, Hermann P, Perske C, Crozier TA, Brück W, et al. (**2011**). Acute effects of intracranial hypertension and ARDS on pulmonary and neuronal damage: A randomized experimental study in pigs. Intensive Care Medicine 37: 1182-1191.

**Heverhagen JT**, Hahn HK, Wegmann M, Herz U, Shaffer Whitaker CD, Matschl V, et al. (**2004**). Volumetric analysis of mice lungs in a clinical magnetic resonance imaging scanner. Magnetic Resonance Materials in Physics, Biology and Medicine 17: 80-85.

**Hill LL**, Chen DL, Kozlowski J, & Schuster DP (**2004**). Neutrophils and neutrophil products do not mediate pulmonary hemodynamic effects of endotoxin on oleic acid-induced lung injury. Anesth Analg 98: 452-457, table of contents.

**Hirose N**, Lynch DA, Cherniack RM, & Doherty DE (**1993**). Correlation between high resolution computed tomography and tissue morphometry of the lung in bleomycin-induced pulmonary fibrosis in the rabbit. American Review of Respiratory Disease 147: 730-738.

**Hodono S**, Shimokawa A, Stewart N. J, Yamauchi Y, Nishimori R, Yamane M, et al. (**2018**). Ethyl Pyruvate Improves Pulmonary Function in Mice with Bleomycin-induced Lung Injury as Monitored with Hyperpolarized 129Xe MR Imaging. Magn Reson Med Sci. 17(4):331-337.

**Horst K**, Simon T. P, Pfeifer R, Teuben M, Almahmoud K, Zhi Q, et al. (**2016**). Characterization of blunt chest trauma in a long-term porcine model of severe multiple trauma. Scientific reports 6:39659.

**Hubner RH**, Gitter W, El Mokhtari NE, Mathiak M, Both M, Bolte H, et al. (**2008**). Standardized quantification of pulmonary fibrosis in histological samples. Biotechniques 44: 507-511, 514-507.

**Ishii T**, Hosoki K, Nikura Y, Yamashita N, Nagase T, & Yamashita N (**2017**). IFN Regulatory Factor 3 Potentiates Emphysematous Aggravation by Lipopolysaccharide. Journal of immunology (Baltimore, Md : 1950) 198: 3637-3649.

**Jacob RE**, Amidan BG, Soelberg J, & Minard KR (**2010**). In vivo MRI of altered proton signal intensity and T2 relaxation in a bleomycin model of pulmonary inflammation and fibrosis. Journal of Magnetic Resonance Imaging 31: 1091-1099.

**Jailkhani N**, Ingram J. R, Rashidian M, Rickelt S, Tian C, Mak H, et al. (**2019**). Noninvasive imaging of tumor progression, metastasis, and fibrosis using a nanobody targeting the extracellular matrix. Proc Natl Acad Sci USA. 116(28):14181-14190.

**Jin GY**, Bok SM, Han YM, Chung MJ, Yoon KH, Kim SR, et al. (**2012**). Effectiveness of rosiglitazone on bleomycin-induced lung fibrosis: Assessed by micro-computed tomography and pathologic scores. Eur J Radiol 81: 1901-1906.

**John AE**, Luckett JC, Tatler AL, Awais RO, Desai A, Habgood A, et al. (**2013**). Preclinical SPECT/CT imaging of alphavbeta6 integrins for molecular stratification of idiopathic pulmonary fibrosis. J Nucl Med 54: 2146-2152.

**Johnson KA** (**2007**). Imaging techniques for small animal imaging models of pulmonary disease: Micro-CT. Toxicologic Pathology 35: 59-64.

**Jones HA**, Clark RJ, Rhodes CG, Schofield JB, Krausz T, & Haslett C (**1994**). In vivo measurement of neutrophil activity in experimental lung inflammation. American Journal of Respiratory and Critical Care Medicine 149: 1635-1639.

**Jones HA**, Schofield JB, Krausz T, Boobis AR, & Haslett C (**1998**). Pulmonary fibrosis correlates with duration of tissue neutrophil activation. American Journal of Respiratory and Critical Care Medicine 158: 620-628.

**Jones HA**, Valind SO, Clark IC, Bolden GE, Krausz T, Schofield JB, et al. (**2002**). Kinetics of lung macrophages monitored in vivo following particulate challenge in rabbits. Toxicol Appl Pharmacol 183: 46-54.

**Karmouty-Quintana H**, Cannet C, Zurbruegg S, Blé FX, Fozard JR, Page CP, et al. (**2007**). Bleomycin-induced lung injury assessed noninvasively and in spontaneously breathing rats by proton MRI. Journal of Magnetic Resonance Imaging 26: 941-949.

**Kaya GC**, Ertay T, Tuna B, Bekis R, Tasci C, Sayit E, et al. (**2006**). Technetium-99m hexamethylpropylene amine oxime lung scintigraphy findings in low-dose amiodarone therapy. Lung 184: 57-61.

**Kelderhouse LE**, Mahalingam S, & Low PS (**2016**). Predicting Response to Therapy for Autoimmune and Inflammatory Diseases Using a Folate Receptor-Targeted Near-Infrared Fluorescent Imaging Agent. Mol Imaging Biol 18: 201-208.

**Kelderhouse LE**, Robins MT, Rosenbalm KE, Hoylman EK, Mahalingam S, & Low PS (**2015**). Prediction of Response to Therapy for Autoimmune/Inflammatory Diseases Using an Activated Macrophage-Targeted Radioimaging Agent. Mol Pharm 12: 3547-3555.

**Kemeny A**, Cseko K, Szitter I, Varga ZV, Bencsik P, Kiss K, et al. (**2017**). Integrative characterization of chronic cigarette smoke-induced cardiopulmonary comorbidities in a mouse model. Environ Pollut 229: 746-759.

**Kersjes W**, Hildebrandt G, Cagil H, Schunk K, Von Zitzewitz H, & Schild H (**1999**). Differentiation of alveolitis and pulmonary fibrosis in rabbits with magnetic resonance imaging after intrabronchial administration of bleomycin. Investigative Radiology 34: 13-21.

**Kimura T**, Nojiri T, Hosoda H, Shintani Y, Inoue M, Miyazato M, et al. (**2015**). Exacerbation of bleomycin-induced injury by lipopolysaccharide in mice: Establishment of a mouse model for acute exacerbation of interstitial lung diseases. European Journal of Cardio-thoracic Surgery 48: e85-e91.

**Kobayashi K**, Horikami D, Omori K, Nakamura T, Yamazaki A, Maeda S, et al. (**2016**). Thromboxane A2 exacerbates acute lung injury via promoting edema formation. Sci Rep. 6:32109.

**Kodama H**, Ueshima E, Gao S, Monette S, Paluch L.R, Howk K, et al. (**2018**). High power microwave ablation of normal swine lung: impact of duration of energy delivery on adverse event and heat sink effects. Int J Hyperthermia. 34(8):1186-1193.

**Komissarov AA**, Florova G, Azghani AO, Buchanan A, Bradley WM, Schaefer C, et al. (**2015**). The time course of resolution of adhesions during fibrinolytic therapy in tetracycline-induced pleural injury in rabbits. American Journal of Physiology - Lung Cellular and Molecular Physiology 309: L562-L572.

**Kwon WJ**, Kim HJ, Jeong YJ, Lee CH, Kim KI, Kim YD, et al. (**2011**). Direct lipiodol injection used for a radio-opaque lung marker: stability and histopathologic effects. Exp Lung Res 37: 310-317.

**Kyriazis A**, Rodriguez I, Nin N, Izquierdo-Garcia JL, Lorente JA, Perez-Sanchez JM, et al. (**2012**). Dynamic ventilation 3 He MRI for the quantification of disease in the rat lung. IEEE Transactions on Biomedical Engineering 59: 777-786.

**Lee HJ**, Goo JM, Kim NR, Kim MA, Chung DH, Son KR, et al. (**2008**). Semiquantitative measurement of murine bleomycin-induced lung fibrosis in in vivo and postmortem conditions using microcomputed tomography: correlation with pathologic scores--initial results. Invest Radiol 43: 453-460.

**Lee JG**, Shim S, Kim MJ, Myung JK, Jang WS, Bae CH, et al. (**2017**). Pentoxifylline Regulates Plasminogen Activator Inhibitor-1 Expression and Protein Kinase A Phosphorylation in Radiation-Induced Lung Fibrosis. BioMed Research International 2017.

**Li M**, Abdollahi A, Grone HJ, Lipson KE, Belka C, & Huber PE (**2009**). Late treatment with imatinib mesylate ameliorates radiation-induced lung fibrosis in a mouse model. Radiat Oncol 4: 66.

**Liu Q**, Ning J, Zhang Y, Wu X, Luo X, & Fan Z (**2010**). Idiopathic pneumonia syndrome in mice after allogeneic bone marrow transplantation: Association between idiopathic pneumonia syndrome and acute graft-versus-host disease. Transplant Immunology 23: 12-17.

**Lynch DA**, Hirose N, Cherniack RM, & Doherty DE (**1997**). Bleomycin-induced lung disease in an animal model: correlation between computed tomography-determined abnormalities and lung function. Academic radiology 4: 102-107.

**Ma H**, Huang D, Zhang M, Huang X, Ma S, Mao S, et al. (**2015**). Lung ultrasound is a reliable method for evaluating extravascular lung water volume in rodents. BMC anesthesiology 15: 162.

**MacVittie T.J**, Farese, A. M, Parker G. A, Jackson W. (**2019**). The Time Course of Radiation-induced Lung Injury in a Nonhuman Primate Model of Partial-body Irradiation with Minimal Bone Marrow Sparing: Clinical and Radiographic Evidence and the Effect of Neupogen Administration. Health Phys. 116(3):366-382.

**Macvittie T.J**, Gibbs A, Farese AM, Barrow K, Bennett A, Taylor-Howell C, et al. (**2017**). AEOL 10150 mitigates radiation-induced lung injury in the nonhuman primate: Morbidity and mortality are administration schedule-dependent. Radiation Research 187: 298-318.

**Major A**, O’Halloran C, Holmes A, Lalor S, Littler R, Spence S, et al. (**2018**). Use of computed tomography imaging during long-term follow-up of nine feline tuberculosis cases. Case Reports J Feline Med Surg. 20(2):189-199.

**Medhora M**, Haworth S, Liu Y, Narayanan J, Gao F, Zhao M, et al. (**2016**). Biomarkers for Radiation Pneumonitis Using Noninvasive Molecular Imaging. J Nucl Med 57: 1296-1301.

**Mendes R.D.S**, Oliveira M.V, Padilha G.A, Rocha N.N, Santos C.L, Maia L.A, et al. (**2019**). Effects of crystalloid, hyper-oncotic albumin, and iso-oncotic albumin on lung and kidney damage in experimental acute lung injury. Respir Res 20(1):155.

**Mokhber Dezfouli M.R**, Jabbari Fakhr M, Sadeghian Chaleshtori S, Dehghan M.M, Vajhi A, Mokhtari R. (**2018**). Intrapulmonary autologous transplant of bone marrow-derived mesenchymal stromal cells improves lipopolysaccharide-induced acute respiratory distress syndrome in rabbit. Crit Care 22(1):353.

**Nagatani Y**, Nitta N, Otani H, Mukaisho K, Sonoda A, Nitta-Seko A, et al. (**2011**). Quantitative Measurement of Bleomycin-induced Lung Fibrosis in Rabbits Using Sequential in vivo Regional Analysis and High-Resolution Computed Tomography: Correlation with Pathologic Findings. Academic Radiology 18: 672-681.

**Nam SW**, Chen X, Lim J, Kim SH, Kim ST, Cho YH, et al. (**2011**). In vivo fluorescence imaging of Bacteriogenic Cyanide in the lungs of live mice infected with cystic fibrosis pathogens. PLoS ONE 6.

**Napp J**, Andrea Markus M, Heck J.G, Dullin C, Möbius W, Gorpas D, et al. (**2018**). Therapeutic fluorescent hybrid nanoparticles for traceable delivery of glucocorticoids to inflammatory sites. Theranostics. 8(22):6367-6383.

**Ning J**, Liu QF, Luo XD, Fan ZP, & Zhang Y (**2009**). Effect and mechanism of acute graft versus host disease on early diffuse murine lung injury following allogeneic stem cell transplantation. Science in China, Series C: Life Sciences 52: 1016-1022.

**Olsson LE**, Smailagic A, Önnervik PO, Lindén A, & Hockings PD (**2011**). 1H and hyperpolarized 3He magnetic resonance imaging clearly detect the preventative effect of a glucocorticoid on endotoxin-induced pulmonary inflammation in vivo. Innate Immunity 17: 204-211.

**Paffett ML**, Hesterman J, Candelaria G, Lucas S, Anderson T, Irwin D, et al. (**2012**). Longitudinal in vivo SPECT/CT imaging reveals morphological changes and cardiopulmonary apoptosis in a rodent model of pulmonary arterial hypertension. PLoS ONE 7.

**Petraitis V**, Petraitiene R, Solomon J, Kelaher AM, Murray HA, Mya-San C, et al. (**2006**). Multidimensional volumetric imaging of pulmonary infiltrates for measuring therapeutic response to antifungal therapy in experimental invasive pulmonary aspergillosis. Antimicrobial Agents and Chemotherapy 50: 1510-1517.

**Povedano J.M**, Martinez P, Serrano R, Tejera A, Gómez-López G, Bobadilla M, et al. (**2018**). Therapeutic effects of telomerase in mice with pulmonary fibrosis induced by damage to the lungs and short telomeres. eLIFE. 7:e31299.

**Qin W**, Liu B, Yi M, Li L, Tang Y, Wu B, et al. (**2018**). Antifibrotic Agent Pirfenidone Protects against Development of Radiation-Induced Pulmonary Fibrosis in a Murine Model. Radiat Res. 190(4):396-403.

**Quintana HK**, Cannet C, Zurbruegg S, Ble FX, Fozard JR, Page CP, et al. (**2006**). Proton MRI as a noninvasive tool to assess elastase-induced lung damage in spontaneously breathing rats. Magn Reson Med 56: 1242-1250.

**Richard JC**, Pouzot C, Gros A, Tourevieille C, Lebars D, Lavenne F, et al. (**2009**). Electrical impedance tomography compared to positron emission tomography for the measurement of regional lung ventilation: an experimental study. Crit Care 13: R82.

**Rooney MB**, & Monnet E (**2002**). Medical and surgical treatment of pyothorax in dogs: 26 Cases (1991-2001). Journal of the American Veterinary Medical Association 221: 86-92.

**Rosen B.H**, Evans T.I.A, Moll S.R, Gray J.S, Liang B, Sun X, et al. (**2018**). Infection is not required for mucoinflammatory lung disease in CFTR-Knockout ferrets. Am J Respir Crit Care Med. 197(10):1308-1318.

**Ruscitti F**, Ravanetti F, Donofrio G, Ridwan Y, van Heijningen P, Essers J, et al. (**2018**). A Multimodal Imaging Approach Based on Micro-CT and Fluorescence Molecular Tomography for Longitudinal Assessment of Bleomycin-Induced Lung Fibrosis in Mice. Journal of visualized experiments. (134):56443.

**Ruscitti F**, Ravanetti F, Essers J, Ridwan Y, Belenkov S, Vos W, et al. (**2017**). Longitudinal assessment of bleomycin-induced lung fibrosis by Micro-CT correlates with histological evaluation in mice. Multidisciplinary Respiratory Medicine 12: 1-10.

**Sakai H**, Horiguchi M, Akita T, Ozawa C, Hirokawa M, Oiso Y, et al. (**2017**). Effect of 4-[(5,6,7,8-Tetrahydro-5,5,8,8-Tetramethyl-2-Naphthalenyl)Carbamoyl]Benzoic Acid (Am80) on Alveolar Regeneration in Adiponectin Deficient-Mice Showing a Chronic Obstructive Pulmonary Disease-Like Pathophysiology. J Pharmacol Exp Ther 361: 501-505.

**Schniering J**, Benešová M, Brunner M, Haller S, Cohrs S, Frauenfelder T, Vrugt B., et al. (**2019**). Visualisation of interstitial lung disease by molecular imaging of integrin αvβ3 and somatostatin receptor 2. Annals of the Rheumatic Diseases 78:2 218-227.

**Schniering J,** Borgna F, Siwowska K, Benesova M, Cohrs S, Hasler R, et al. (**2018**). In Vivo Labeling of Plasma Proteins for Imaging of Enhanced Vascular Permeability in the Lungs. Mol Pharm. 15(11):4995-5004.

**Schniering J,** Guo L, Brunner M, Schibli R, Ye S, Distler O, et al. (**2018**). Evaluation of 99mTc-rhAnnexin V-128 SPECT/CT as a diagnostic tool for early stages of interstitial lung disease associated with systemic sclerosis. Arthritis Res Ther. 16;20(1):183.

**Schueller-Weidekamm C**, Wassermann E, Redl H, Prokop M, Zimpfer M, Herold C, et al. (**2006**). Dynamic CT measurement of pulmonary enhancement in piglets with experimental acute respiratory distress syndrome. Radiology 239: 398-405.

**Schuster DP**, & Howard DK (**1994**). The effect of positive end-expiratory pressure on regional pulmonary perfusion during acute lung injury. Journal of critical care 9: 100-110.

**Schuster DP**, Sandiford P, & Stephenson AH (**1993**). Thromboxane receptor stimulation/inhibition and perfusion redistribution after acute lung injury. Journal of Applied Physiology 75: 2069-2078.

**Schwenke DO**, Pearson JT, Shimochi A, Kangawa K, Tsuchimochi H, Umetani K, et al. (**2009**). Changes in pulmonary blood flow distribution in monocrotaline compared with hypoxia-induced models of pulmonary hypertension: Assessed using synchrotron radiation. Journal of Hypertension 27: 1410-1419.

**Schwenke DO**, Pearson JT, Sonobe T, Ishibashi-Ueda H, Shimouchi A, Kangawa K, et al. (**2011**). Role of Rho-kinase signaling and endothelial dysfunction in modulating blood flow distribution in pulmonary hypertension. Journal of Applied Physiology 110: 901-908.

**Shao R**, Wang F. J, Lyu M, Yang J, Zhang P, Zhu Y. (**2019**). Ability to Suppress TGF-beta-Activated Myofibroblast Differentiation Distinguishes the Anti-pulmonary Fibrosis Efficacy of Two Danshen-Containing Chinese Herbal Medicine Prescriptions. Front Pharmacol. 10:412.

**Sharpe JP**, Khan NR, Chatterjee AR, Huang J, Magnotti LJ, Croce MA, et al. (**2017**). Investigating cyclooxygenase inhibition in a rat pulmonary contusion model: A laboratory study finding no improvement with ibuprofen. American Surgeon 83: 666-672.

**Shea B. S**, Probst C. K, Brazee P. L., Rotile N. J, Blasi F, Weinreb P. H, et al. (**2017**). Uncoupling of the profibrotic and hemostatic effects of thrombin in lung fibrosis. JCI Insight. 2(9):e86608.

**Shioya S**, Christman R, Ailion DC, Cutillo AG, & Goodrich KC (**1993**). Nuclear magnetic resonance Hahn spin-echo decay (T2) in live rats with endotoxin lung injury. Magnetic Resonance in Medicine 29: 441-445.

**Shofer S**, Badea C, Auerbach S, Schwartz DA, & Johnson GA (**2007**). A micro-computed tomography-based method for the measurement of pulmonary compliance in healthy and bleomycin-exposed mice. Experimental Lung Research 33: 169-183.

**Shofer S**, Badea C, Qi Y, Potts E, Foster WM, & Johnson GA (**2008**). A micro-CT analysis of murine lung recruitment in bleomycin-induced lung injury. J Appl Physiol (1985) 105: 669-677.

**Shu HKG**, Yoon Y, Hong S, Xu K, Gao H, Hao C, et al. (**2013**). Inhibition of the CXCL12/CXCR4-axis as preventive therapy for radiation-induced pulmonary fibrosis. PLoS ONE 8.

**Sonoda A**, Nitta N, Tsuchiya K, Otani H, Watanabe S, Mukaisho K, et al. (**2014**). Asialoerythropoietin ameliorates bleomycin-induced acute lung injury in rabbits by reducing inflammation. Experimental and therapeutic medicine 8: 1443-1446.

**Stellari F**, Bergamini G, Ruscitti F, Sandri A, Ravanetti F, Donofrio G, et al. (**2016**). In vivo monitoring of lung inflammation in CFTR-deficient mice. Journal of Translational Medicine 14.

**Stellari F**, Bergamini G, Sandri A, Donofrio G, Sorio C, Ruscitti F, et al. (**2015**). In vivo imaging of the lung inflammatory response to Pseudomonas aeruginosa and its modulation by azithromycin. Journal of Translational Medicine 13.

**Stellari FF**, Ruscitti F, Pompilio D, Ravanetti F, Tebaldi G, Macchi F, et al. (**2017**). Heterologous matrix metalloproteinase gene promoter activity allows in vivo real-time imaging of bleomycin-induced lung fibrosis in transiently transgenized mice. Frontiers in Immunology 8.

**Stephen MJ**, Emami K, Woodburn JM, Chia E, Kadlecek S, Zhu J, et al. (**2010**). Quantitative assessment of lung ventilation and microstructure in an animal model of idiopathic pulmonary fibrosis using hyperpolarized gas MRI. Acad Radiol 17: 1433-1443.

**Strobel K**, Hoerr V, Schmid F, Wachsmuth L, Loffler B, & Faber C (**2012**). Early detection of lung inflammation: exploiting T1-effects of iron oxide particles using UTE MRI. Magn Reson Med 68: 1924-1931.

**Suga K**, Uchisako H, Nishigauchi K, Shimizu K, Kume N, Yamada N, et al. (**1994**). Technetium-99m-HMPAO as a marker of chemical and irradiation lung injury: Experimental and clinical investigations. Journal of Nuclear Medicine 35: 1520-1527.

**Suga K**, Yuan Y, Ogasawara N, Tsukuda T, & Matsunaga N (**2003**). Altered clearance of gadolinium diethylenetriaminepentaacetic acid aerosol from bleomycin-injured dog lungs: Initial observations. American Journal of Respiratory and Critical Care Medicine 167: 1704-1710.

**Tassali N**, Bianchi A, Lux F, Raffard G, Sanchez S, Tillement O, et al. (**2016**). MR imaging, targeting and characterization of pulmonary fibrosis using intra-tracheal administration of gadolinium-based nanoparticles. Contrast Media and Molecular Imaging 11: 396-404.

**Thomas AC**, Nouls JC, Driehuys B, Voltz JW, Fubara B, Foley J, et al. (**2011**). Ventilation defects observed with hyperpolarized 3He magnetic resonance imaging in a mouse model of acute lung injury. American Journal of Respiratory Cell and Molecular Biology 44: 648-654.

**Tigani B**, Cannet C, Zurbrügg S, Schaeublin E, Mazzoni L, Fozard JR, et al. (**2003**). Resolution of the oedema associated with allergic pulmonary inflammation in rats assessed noninvasively by magnetic resonance imaging. British Journal of Pharmacology 140: 239-246.

**Tournebize R**, Doan BT, Dillies MA, Maurin S, Beloeil JC, & Sansonetti PJ (**2006**). Magnetic resonance imaging of Klebsiella pneumoniae-induced pneumonia in mice. Cellular Microbiology 8: 33-43.

**van Berlo D**, Khmelinskii A, Gasparini A, Salguero F. J, Floot B, de Wit N, et al. (**2019**). Micro cone beam computed tomography for sensitive assessment of radiation-induced late lung toxicity in preclinical models. Radiother Oncol. 138:17-24.

**Van Heerde M**, Roubik K, Kopelent V, Kneyber MCJ, & Markhorst DG (**2010**). Spontaneous breathing during high-frequency oscillatory ventilation improves regional lung characteristics in experimental lung injury. Acta Anaesthesiologica Scandinavica 54: 1248-1256.

**Vande Velde G**, Poelmans J, De Langhe E, Hillen A, Vanoirbeek J, Himmelreich U, et al. (**2016**). Longitudinal micro-CT provides biomarkers of lung disease that can be used to assess the effect of therapy in preclinical mouse models, and reveal compensatory changes in lung volume. Disease models & mechanisms 9: 91-98.

**Vande Velde G**, De Langhe E, Poelmans J, Bruyndonckx P, d'Agostino E, Verbeken E, et al. (**2015**). Longitudinal in vivo microcomputed tomography of mouse lungs: No evidence for radiotoxicity. Am J Physiol Lung Cell Mol Physiol 309: L271-279.

**Vande Velde G**, De Langhe E, Poelmans J, Dresselaers T, Lories RJ, & Himmelreich U (**2014**). Magnetic resonance imaging for noninvasive assessment of lung fibrosis onset and progression: cross-validation and comparison of different magnetic resonance imaging protocols with micro-computed tomography and histology in the bleomycin-induced mouse model. Invest Radiol 49: 691-698.

**Velten M**, Britt Jr RD, Heyob KM, Welty SE, Eiberger B, Tipple TE, et al. (**2012**). Prenatal inflammation exacerbates hyperoxia-induced functional and structural changes in adult mice. American Journal of Physiology - Regulatory Integrative and Comparative Physiology 303: 279-290.

**Via LE**, Schimel D, Weiner DM, Dartois V, Dayao E, Cai Y, et al. (**2012**). Infection dynamics and response to chemotherapy in a rabbit model of tuberculosis using [18F]2-fluoro-deoxy-D-glucose positron emission tomography and computed tomography. Antimicrobial Agents and Chemotherapy 56: 4391-4402.

**Vij N**, Min T, Marasigan R, Belcher CN, Mazur S, Ding H, et al. (**2010**). Development of PEGylated PLGA nanoparticle for controlled and sustained drug delivery in cystic fibrosis. Journal of Nanobiotechnology 8.

**Waerhaug K**, Kuzkov VV, Kuklin VN, Mortensen R, Nordhus KC, Kirov MY, et al. (**2009**). Inhaled aerosolised recombinant human activated protein C ameliorates endotoxin-induced lung injury in anaesthetised sheep. Crit Care 13: R51.

**Waghorn P. A**, Jones C. M, Rotile N. J, Koerner S. K, Ferreira D. S, Chen H. H, et al. (**2017**). Molecular Magnetic Resonance Imaging of Lung Fibrogenesis with an Oxyamine-Based Probe. Angew Chem Int Ed Engl. 56(33):9825-9828.

**Ward WF**, Lin PJP, Wong PS, Behnia R, & Jalali N (**1993**). Radiation pneumonitis in rats and its modification by the angiotensin- converting enzyme inhibitor captopril evaluated by high-resolution computed tomography. Radiation Research 135: 81-87.

**Watanabe S**, Nitta N, Sonoda A, Nitta-Seko A, Ohta S, Tsuchiya K, et al. (**2013**). Inhibition of fibrosis and inflammation by triple therapy with pirfenidone, edaravone and erythropoietin in rabbits with drug-induced lung injury: Comparison of CT imaging and pathological findings. Experimental and therapeutic medicine 6: 1096-1100.

**Wehner AP** (**1980**). Effects of inhaled asbestos, asbestos plus cigarette smoke, asbestos-cement and talc baby powder in hamsters. IARC scientific publications: 373-376.

**Weiner RE**, Sasso DE, Gionfriddo MA, Thrall RS, Syrbu S, Smilowitz HM, et al. (**2001**). Early detection of oleic acid-induced lung injury in rats using 111In-labeled anti-rat intercellular adhesion molecule-1. Journal of Nuclear Medicine 42: 1109-1115.

**Weiner RE**, Sasso DE, Gionfriddo MA, Syrbu SI, Smilowitz HM, Vento J, et al. (**1998**). Early detection of bleomycin-induced lung injury in rat using indium- 111-labeled antibody directed against intercellular adhesion molecule-1. Journal of Nuclear Medicine 39: 723-728.

**Weishaupt D**, Hilfiker PR, Schmidt M, & Debatin JF (**1999**). Pulmonary hemorrhage: imaging with a new magnetic resonance blood pool agent in conjunction with breathheld three-dimensional magnetic resonance angiography. Cardiovascular and interventional radiology 22: 321-325.

**Wenlong L**, Leilei Y, Wei F, Yi C, Jing T, Lanzhi M, et al. (**2015**). Luciferase expression is driven by the promoter of fibroblast activation protein-α in murine pulmonary fibrosis. Biotechnology letters 37: 1757-1763.

**Withana NP**, Ma X, McGuire HM, Verdoes M, van der Linden WA, Ofori LO, et al. (**2016**). Non-invasive Imaging of Idiopathic Pulmonary Fibrosis Using Cathepsin Protease Probes. Sci Rep 6: 19755.

**Xiang B**, Chen L, Wang X, Zhao Y, Wang Y, & Xiang C (**2017**). Transplantation of Menstrual Blood-Derived Mesenchymal Stem Cells Promotes the Repair of LPS-Induced Acute Lung Injury. Int J Mol Sci 18.

**Xiong Y,** Nie D, Liu S, Ma H, Su S, Sun A, et al. (**2019**). Apoptotic PET Imaging of Rat Pulmonary Fibrosis with Small-Molecule Radiotracer. Mol Imaging Biol 21(3):491-499.

**Xiong Y**, Nie D, Liu S, Ma H, Su S, Sun A, et al. (**2018**). Apoptotic PET Imaging of Rat Pulmonary Fibrosis with [18F]ML-8. Crit Care 22(1):353.

**Xu Y**, Ge L, Abdel-Razek O, Jain S, Liu Z, Hong Y, et al. (**2016**). Differential Susceptibility of Human Sp-B Genetic Variants on Lung Injury Caused by Bacterial Pneumonia and the Effect of a Chemically Modified Curcumin. Shock 45: 375-384.

**Yangi B**, Cengiz Ustuner M, Dincer M, Ozbayer C, Tekin N, Ustuner D, et al. (**2018**). Propolis protects endotoxin induced acute lung and liver inflammation through attenuating inflammatory responses and oxidative stress. J Med Food. 21(11):1096-1105.

**Yen S**, Preissner M, Bennett E, Dubsky S, Carnibella R, O’Toole R, et al. (**2019**). The link between regional tidal stretch and lung injury during mechanical ventilation. Am J Respir Cell Mol Biol. 60(5):569-577.

**Yu W**, Mi L, & Long T (**2017**). Efficacies of rosiglitazone and retinoin on bleomycin-induced pulmonary fibrosis in rats. Experimental and therapeutic medicine 14: 609-615.

**Yu WC**, Tian LY, & Cheng W (**2015**). Efficacy study of edaravone and acetylcysteine towards bleomycin-induced rat pulmonary fibrosis. International Journal of Clinical and Experimental Medicine 8: 8730-8739.

**Zhao L**, Ashek A, Wang L, Fang W, Dabral S, Dubois O, et al. (**2013**). Heterogeneity in lung (18)FDG uptake in pulmonary arterial hypertension: potential of dynamic (18)FDG positron emission tomography with kinetic analysis as a bridging biomarker for pulmonary vascular remodeling targeted treatments. Circulation 128: 1214-1224.

**Zhu X**, Cao W, Chang B, Zhang L, Qiao P, Li X, et al. (**2016**). Polyacrylate/nanosilica causes pleural and pericardial effusion, and pulmonary fibrosis and granuloma in rats similar to those observed in exposed workers. International journal of nanomedicine 11: 1593-1605.

**Zurek M**, Boyer L, Caramelle P, Boczkowski J, & Crémillieux Y (**2012**). Longitudinal and noninvasive assessment of emphysema evolution in a murine model using proton MRI. Magnetic Resonance in Medicine 68: 898-904.
